# Continuous Hesitant Fuzzy Aggregation Operators and Their Application to Decision Making under Interval-Valued Hesitant Fuzzy Setting

**DOI:** 10.1155/2014/897304

**Published:** 2014-05-25

**Authors:** Ding-Hong Peng, Tie-Dan Wang, Chang-Yuan Gao, Hua Wang

**Affiliations:** ^1^Institute of Quality Development, Kunming University of Science and Technology, Kunming 650093, China; ^2^School of Management, Harbin University of Science and Technology, Harbin 150040, China

## Abstract

Interval-valued hesitant fuzzy set (IVHFS), which is the further generalization of hesitant fuzzy set, can overcome the barrier that the precise membership degrees are sometimes hard to be specified and permit the membership degrees of an element to a set to have a few different interval values. To efficiently and effectively aggregate the interval-valued hesitant fuzzy information, in this paper, we investigate the continuous hesitant fuzzy aggregation operators with the aid of continuous OWA operator; the C-HFOWA operator and C-HFOWG operator are presented and their essential properties are studied in detail. Then, we extend the C-HFOW operators to aggregate multiple interval-valued hesitant fuzzy elements and then develop the weighted C-HFOW (WC-HFOWA and WC-HFOWG) operators, the ordered weighted C-HFOW (OWC-HFOWA and OWC-HFOWG) operators, and the synergetic weighted C-HFOWA (SWC-HFOWA and SWC-HFOWG) operators; some properties are also discussed to support them. Furthermore, a SWC-HFOW operators-based approach for multicriteria decision making problem is developed. Finally, a practical example involving the evaluation of service quality of high-tech enterprises is carried out and some comparative analyses are performed to demonstrate the applicability and effectiveness of the developed approaches.

## 1. Introduction

As a novel generalization of fuzzy sets, hesitant fuzzy sets (HFSs) [1, 2] introduced by Torra and Narukawa have been successfully used in the decision making field as a powerful tool for processing uncertain and vague information. Different from the other generalizations of fuzzy sets, HFSs permit the membership degree of an element to a set to be represented as several possible values between 0 and 1, which are very useful in dealing with the situations where people hesitate between several values to express their judgments [3–5] or their opinions with incongruity [6–8], particularly, the group decision making with anonymity [9–12]. Meanwhile, HFSs can also avoid performing information aggregation and can directly reflect the differences of the opinions of different experts [1, 13, 14]. Furthermore, as Torra reported that the envelope of HFS is an intuitionistic fuzzy set (IFS), all HFSs are type-2 fuzzy sets, and HFSs and fuzzy multisets (FMSs) have the same form, but their operations are different [2]. Thus HFSs open new views for further research on decision making under hesitant environments and have received much attention from many authors. Torra and Narukawa [1, 2] proposed some set theoretic operations such as union, intersection and complement, and the extension principle on HFSs. Subsequently, Xia and Xu [6] defined some new operations on HFSs based on the interconnection between HFSs and IFSs and then made an intensive study of hesitant fuzzy information aggregation techniques and their applications in decision making. Xu and Xia [7] investigated some distance measures for HFSs drawing on the well-known Hamming distance, the Euclidean distance, the Hausdorff metric, and their generalizations. Following these pioneering studies, many subsequent studies on the basic theory [9, 15], the aggregation operators [8, 9, 12–17], the discrimination measures [18] (including distance measures [3–5, 7], similarity measures [7], correlation measures [3, 19], entropy, and cross-entropy [20], etc.) for HFSs, and the further extensions of the HFSs such as the interval-values HFSs (IVHFSs) [11, 19], the dual (or generalized) HFSs (DHFSs) [10, 21, 22], and the hesitant fuzzy linguistic term sets (HFLTSs) [23, 24] have been conducted.

In some practical decision making problems, however, the precise membership degrees of an element to a set are sometimes hard to be specified. To overcome the barrier, Chen et al. [11, 19] proposed the concept of interval-valued hesitant fuzzy sets (IVHFSs) that represent the membership degrees of an element to a set with several possible interval values and then presented some interval-valued hesitant fuzzy aggregation operators. Although the concept of IVHFSs is very recent, it has received a lot of attention by other researchers in the community; Wei [25] presented some interval-valued hesitant fuzzy Choquet ordered averaging operators, interval-valued hesitant fuzzy prioritized aggregation operators, and interval-valued hesitant fuzzy power aggregation operators. Wei and Zhao [26] presented some interval-valued hesitant fuzzy Einstein aggregation operators and induced interval-valued hesitant fuzzy Einstein aggregation operators. Li and Peng [27] presented some interval-valued hesitant fuzzy Hamacher synergetic weighted aggregation operators to select shale gas areas. Peng and Wang [17] presented some dynamic interval-valued hesitant fuzzy aggregation operators to aggregate the interval-valued hesitant fuzzy information collected at different periods in multiperiod decision making. Nevertheless, the above-mentioned operators are straightforward extensions of their respective proposals for the case of HFEs; they only focus on the endpoints of the closed intervals of interval-valued hesitant fuzzy elements (IVHFEs) on the basis of the characteristics of interval numbers and therefore are not rich enough to capture all the information contained in IVHFEs. Additionally, due to the fact that decision making problems are essentially humanistic and subjective in nature, decision makers' (DMs') risk preferences play an important role. How to reflect DMs' risk preferences in decision making is a crucial problem. Yet the above-mentioned operators do not consider the problem. Thus, it is necessary to explore some new techniques for aggregating interval-valued hesitant fuzzy information in accordance with DMs' risk preferences. The continuous ordered weighted averaging (C-OWA) operator was formally presented by Yager [28] (which was previously introduced by Torra and Godo [29, 30] in 1997, see also [31]) and is appropriate for aggregating decision information which is given in the forms of valued interval. A distinguished advantage of C-OWA operator is that it can lead to every value in the interval being aggregated and aggregate the valued interval to a precise value based on decision attitudes of DMs. Thus, based on the C-OWA operator, some extended continuous aggregation operators are further developed, such as the continuous ordered weighted geometric (C-OWG) operator [32], the continuous generalized OWA (C-GOWA) operator [33], the continuous quasi-OWA (C-QOWA) operator [34], and the induced generalized continuous OWA (IGCOWA) operator [35]. in view of the predominant advantages of C-OWA operator, in this paper, we investigate the continuous hesitant fuzzy aggregation operators to efficiently and effectively aggregate the interval-valued hesitant fuzzy information and apply them to the multiple criteria decision making.

To do so, the remainder of this paper is set out as follows.  Section 2 introduces some preliminary concepts including hesitant fuzzy sets, interval-valued hesitant fuzzy sets, and continuous OW (C-OWA and C-OWG) operators. In Section 3, we propose the continuous HFOWA operator; the continuous HFOWG operator and their essential properties are studied in detail. In Section 4, we extend the C-HFOW operators to efficiently and effectively aggregate multiple interval-valued hesitant fuzzy elements and then develop the weighted C-HFOWA operator, the weighted C-HFOWG operator, the ordered weighted C-GOWA operator, the ordered weighted C-GOWG operator, the synergetic weighted C-GOWA operator, and the synergetic weighted C-GOWG operator; some properties are also discussed to support them. In Section 5, we develop an approach based on the SWC-HFOW operators to multicriteria decision-making under interval-valued hesitant fuzzy environments and in Section 6 a practical example involving the evaluation of service quality of high-tech enterprises is carried out and some comparative analyses are performed to demonstrate the applicability and effectiveness of the developed approaches. Finally, we summarize the main conclusions of the paper in Section 7.

## 2. Preliminaries

In this section, we introduce some basic notions related to hesitant fuzzy sets, interval-valued hesitant fuzzy sets, and continuous OW operators.

### 2.1. Hesitant Fuzzy Sets

Hesitant fuzzy sets (HFSs) are quite suited for the situation where we have a set of possible values, rather than a margin of error or some possibility distribution on the possible values. Thus, HFSs can be considered as a powerful tool to express uncertain information in the process of decision making with hesitancy and incongruity.

**Definition 1 (see [2])**

Let *X* be a fixed set; a hesitant fuzzy set (HFS) on *X* is in terms of a function that when applied to *X* returns a subset of [0, 1].

To be easily understood, Xia and Xu [6] expressed the HFS as the following mathematical symbol:

(1) $$\begin{array}{c} E=\left\{\langle x,h_{E}\left(x\right)\rangle \;|\;x\in X\right\}, \end{array}$$

where *h*_*E*_(*x*) is a set of values in [0, 1], denoting the possible membership degrees of the element *x* ∈ *X* to the set *E*. For convenience, we call *h*_*E*_(*x*) a hesitant fuzzy element (HFE).

**Definition 2 (see [6])**

Let *h*, *h*_1_, and *h*_2_ be three HFEs; then

1.  
*h* ^*λ*^ = ⋃_*γ*∈*h*_{*γ* ^*λ*^}
2. *λh* = ⋃_*γ*∈*h*_{1 − (1 − *γ*)^*λ*^}
3. *h*_1_ ⊕ *h*_2_ = ⋃_*γ*_1_∈*h*_1_,*γ*_2_∈*h*_2__{*γ*_1_ + *γ*_2_ − *γ*_1_ *γ*_2_}
4. *h*_1_ ⊗ *h*_2_ = ⋃_*γ*_1_∈*h*_1_,*γ*_2_∈*h*_2__{*γ*_1_ *γ*_2_}.

To compare the HFEs, Xia and Xu [6] defined the following comparison laws.

**Definition 3 (see [6])**

For a HFE *h*, *s*(*h*) = (1/#*h*)∑_*γ*∈*h*_ *γ* is called the score function of *h*, where #*h* is the number of the values in *h*. Moreover, for two HFEs *h*_1_ and *h*_2_, if *s*(*h*_1_) > *s*(*h*_2_), then *h*_1_ > *h*_2_; if *s*(*h*_1_) = *s*(*h*_2_), then *h*_1_ = *h*_2_.

It is noted that the numbers of values in different HFEs may be different, and thus the traditional operations and operators cannot be used. For the aggregation of hesitant fuzzy information, Torra and Narukawa [1] proposed the following extension principle that extends functions to HFEs.

**Definition 4 (see [1])**

Let *E* = {*h*_1_, *h*_2_,…, *h*_*n*_} be a set of *n* HFEs and let Θ be a function on *E*, Θ : [0,1]^*N*^ → [0,1]; then

(2) $$\begin{array}{c} \Theta_{E}=\underset{\gamma \in \{h_{1}\times h_{2}\times \cdots \times h_{n}\}}{⋃}\left\{\Theta \left(\gamma \right)\right\}. \end{array}$$

Through the extension principle, one can not only realize the synthesis of HFEs with different numbers of values but also utilize properly all information in HFEs, and it can guarantee that the properties on Θ lead to related properties on Θ_*E*_, which is also an essential difference between the operations of HFSs and the ones of the FMSs.

Based on the above extension principle, Xia and Xu [6] developed a series of specific aggregation operators for HFEs.

**Definition 5 (see [6])**

Let *h*_*j*_  (*j* = 1,2,…, *n*) be a collection of HFEs, *h*_*σ*(*j*)_ is the *j*th largest of them, and *w* = (*w*_1_, *w*_2_,…, *w*_*n*_) is the associated (order) weight vector with *w*_*i*_ ∈ [0,1] and ∑_*i*=1_^*n*^ *w*_*i*_ = 1, then consider the following.

(1) A hesitant fuzzy ordered weighted averaging (HFOWA) operator is a mapping HFOWA : *H* ^*n*^ → *H*, such that
  (3) HFOWA(h1,h2,…,hn)  =⨁j=1n(wjhσ(j))  =⋃γσ(j)∈hσ(j),j=1,2…,n{1−∏j=1n(1−γσ(j))wj}.
(2) A hesitant fuzzy ordered weighted geometric (HFOWG) operator is a mapping HFOWG : *H* ^*n*^ → *H*, such that
  (4) HFOWG(h1,h2,…,hn)  =⨁j=1n(hσ(j)wj)  =⋃γσ(j)∈hσ(j),j=1,2...,n{∏j=1nγσ(j)wj}.
  The results of the hesitant fuzzy aggregation operators are also HFEs.

### 2.2. Interval-Valued Hesitant Fuzzy Sets

To overcome the barrier that the precise membership degrees of an element to a set are sometimes hard to be specified, Chen et al. [11, 19] introduced the interval-valued hesitant fuzzy sets (IVHFSs) which permit the membership degrees of an element to a set to be several possible interval values.

**Definition 6 (see [11])**

Let *X* be a reference set, and let *D*[0,1] be the set of all closed subintervals of [0,1]; then an IVHFS on *X* is defined as

(5) $$\begin{array}{c} \tilde{E}=\left\{\langle x,{\tilde{h}}_{\tilde{E}}\left(x\right)\rangle \;|\;x\in X\right\}, \end{array}$$

where ${\tilde{h}}_{\tilde{E}}(x)$: *x* → *D*[0,1] denotes all possible interval-valued membership degrees of the element *x* ∈ *X* to the set $\tilde{E}$. For convenience, we call ${\tilde{h}}_{\tilde{E}}\left(x\right)$ an interval-valued hesitant fuzzy element (IVHFE), which reads

(6) $$\begin{array}{c} {\tilde{h}}_{\tilde{E}}\left(x\right)=\underset{\tilde{\gamma }\in {\tilde{h}}_{\tilde{E}}\left(x\right)}{⋃}\left\{\tilde{\gamma }=\left[{\tilde{\gamma }}^{L},{\tilde{\gamma }}^{U}\right]\right\}. \end{array}$$

**Definition 7 (see [11])**

Let $\tilde{h}$, ${\tilde{h}}_{1}$, and ${\tilde{h}}_{2}$ be three IVHFEs; then

1. $\lambda \tilde{h}={⋃}_{\tilde{\gamma }\in \tilde{h}}\{[1-{\left(1-{\tilde{\gamma }}^{L}\right)}^{\lambda },1-{\left(1-{\tilde{\gamma }}^{U}\right)}^{\lambda }]\}$,
2. ${\tilde{h}}^{\lambda }={⋃}_{\tilde{\gamma }\in \tilde{h}}\left\{[{\left({\tilde{\gamma }}^{L}\right)}^{\lambda },{\left({\tilde{\gamma }}^{U}\right)}^{\lambda }]\right\}$,
3. ${\tilde{h}}_{1}⊕{\tilde{h}}_{2}={⋃}_{{\tilde{\gamma }}_{1}\in {\tilde{h}}_{1},{\tilde{\gamma }}_{2}\in {\tilde{h}}_{2}}\left\{({\tilde{\gamma }}_{1}^{L}+{\tilde{\gamma }}_{2}^{L}-{\tilde{\gamma }}_{1}^{L}{\tilde{\gamma }}_{2}^{L},{\tilde{\gamma }}_{1}^{U}+{\tilde{\gamma }}_{2}^{U}-{\tilde{\gamma }}_{1}^{U}{\tilde{\gamma }}_{2}^{U})\right\}$,
4. ${\tilde{h}}_{1}⊗{\tilde{h}}_{2}={⋃}_{{\tilde{\gamma }}_{1}\in {\tilde{h}}_{1},{\tilde{\gamma }}_{2}\in {\tilde{h}}_{2}}\left\{[{\tilde{\gamma }}_{1}^{L}{\tilde{\gamma }}_{2}^{L},{\tilde{\gamma }}_{1}^{U}{\tilde{\gamma }}_{2}^{U}]\right\}$.

Chen et al. [11] defined the score function of IVHFE and utilized the possibility degree formula to compare the score values of two IVHFEs.

**Definition 8**

For an IVHFE $\tilde{h}$, $s\left(\tilde{h}\right)=(1/\#\tilde{h})\sum_{\tilde{\gamma }\in \tilde{h}}\tilde{\gamma }=\left[(1/\#\tilde{h})\sum_{\tilde{\gamma }\in \tilde{h}}{\tilde{\gamma }}^{L},(1/\#\tilde{h})\sum_{\tilde{\gamma }\in \tilde{h}}{\tilde{\gamma }}^{U}\right]$ is called the score function of $\tilde{h}$. Moreover, for two IVHFEs *h*_1_ and *h*_2_, if

(7) P(h~1≥h~2)=max⁡{1−max⁡((1#h~2∑γ~2∈h~2γ~2U−1#h~1∑γ~1∈h~1γ~1L)  ×(1#h~1∑γ~1∈h~1(γ~1U−γ~1L) +1#h~2∑γ~2∈h~2(γ~2U−γ~2L))−1,0)0}>0.5,

then ${\tilde{h}}_{1}>{\tilde{h}}_{2}$; if $P({\tilde{h}}_{1}\geq {\tilde{h}}_{2})=0.5$, then ${\tilde{h}}_{1}={\tilde{h}}_{2}$.

**Definition 9 (see [11])**

Let ${\tilde{h}}_{j}\;(j=1,2,\ldots ,n)$ be a collection of IVHFEs, ${\tilde{h}}_{\sigma (j)}$ be the *j*th largest of them, *w* = (*w*_1_, *w*_2_,…, *w*_*n*_) be the associated weight vector with *w*_*i*_ ∈ [0,1], and ∑_*i*=1_^*n*^ *w*_*i*_ = 1, then consider the following.

(1) An interval-valued hesitant fuzzy ordered weighted averaging (IVHFOWA) operator is a mapping $\text{IVHFOWA}:{\tilde{H}}^{n}\to \tilde{H}$, where
  (8) IVHFOWA(h~1,h~2,…,h~n) =⨁j=1n(wjh~σ(j)) =⋃γ~σ(j)∈h~σ(j),j=1,2,…,n{[1−∏j=1n(1−γσ(j)L)wj,1−∏j=1n(1−γσ(j)U)wj]}.
(2) An interval-valued hesitant fuzzy ordered weighted geometric (IVHFOWG) operator is a mapping $\text{IVHFOWG}:{\tilde{H}}^{n}\to \tilde{H}$, where
  (9) IVHFOWG(h~1,h~2,…,h~n)  =⨁j=1n(h~σ(j)wj)  =⋃γ~σ(j)∈h~σ(j),j=1,2,...,n{[∏j=1n(γσ(j)L)wj,∏j=1n(γσ(j)U)wj]}.

The results of the IVHFOWA and IVHFOWG operators are also IVHFEs; that is, the results consist of some interval values. Meanwhile, as the analysis above, the operators only focus on the endpoints of the closed intervals of IVHFEs and therefore are not rich enough to capture all the information contained in IVHFEs. Furthermore, they do not consider the DMs' risk preferences in aggregation process.

### 2.3. Continuous Ordered Weighted Aggregation Operators

**Definition 10 (see [36])**

An OWA operator of dimensions *n* is a mapping OWA : *R* ^*n*^ → *R* that has an associated weight vector *w* = (*w*_1_, *w*_2_,…, *w*_*n*_) with the properties 0 ≤ *w*_*j*_ ≤ 1  (*j* = 1,2,…, *n*) and ∑_*j*=1_^*n*^ *w*_*j*_ = 1, such that

(10) $$\begin{array}{c} \text{OWA}\left(a_{1},a_{2},\ldots ,a_{n}\right)=w_{1}a_{\sigma (1)}+w_{2}a_{\sigma (2)}+\cdots +w_{n}a_{\sigma (1)}, \end{array}$$

where *σ* defines a permutation of {1,2,…, *n*} such that *a*_*σ*(*j*)_ ≥ *a*_*σ*(*j*+1)_ for all *j*.

The OWA operator is bounded, idempotent, commutative, and monotonic. Note that the weights are assigned according to the positions of argument variables in OWA operator, that is, each argument value and its corresponding associated weight existing one-to-one relative relations [4, 27, 37, 38]; thus we can find a permutation *ρ* : {1,2,…, *n*}→{1,2,…, *n*}, which is the inverse permutation of *σ*; that is, *ρ* = *σ* ^−1^, and the OWA operator can be alternatively defined as

(11) $$\begin{array}{c} \text{OW}{\text{A}}^{\prime }\left(a_{1},a_{2},\ldots ,a_{n}\right)=w_{\rho (1)}a_{1}+w_{\rho (2)}a_{2}+\cdots +w_{\rho (n)}a_{n}. \end{array}$$

**Proposition 11**

*w* = (*w*_1_, *w*_2_,…, *w*_*n*_) is the associated weight vector with *w*_*i*_ ∈ [0,1], ∑_*i*=1_^*n*^ *w*_*i*_ = 1, *ρ*(·) and *σ*(·) are two permutations of {1,2,…, *n*}, if *ρ*(·) = *σ*(·)^−1^, then

(12) $$\begin{array}{c} \text{OWA}\left(a_{1},a_{2},\ldots ,a_{n}\right)={\text{OWA}}^{\prime }\left(a_{1},a_{2},\ldots ,a_{n}\right). \end{array}$$

Proposition 11 shows the equivalence between the original definition and the alternative definition of the OWA operator.

In order to aggregate all the values in a closed interval [*a*, *b*], Yager [28] presented a continuous ordered weighted averaging (C-OWA) operator based on OWA operator and the basic unit-interval monotonic (BUM) function [39].

**Definition 12 (see [28])**

A continuous ordered weighted averaging (C-OWA) operator is a mapping *P*_*Q*_^*A*^ : *Ω* ^+^ → *R* ^+^ which is defined as follows:

(13) $$\begin{array}{c} P_{Q}^{A}\left(\left[a,b\right]\right)=\overset{1}{\underset{0}{\int }}\frac{dQ\left(y\right)}{dy}\left[b-\left(b-a\right)y\right]dy, \end{array}$$

where *Q* is a BUM function *Q* : [0,1]→[0,1] and is monotonic with the properties (1)  *Q*(0) = 0, (2)  *Q*(1) = 1, and (3)  *Q*(*x*) ≥ *Q*(*y*) if *x* > *y*. *Ω* ^+^ = {[*a*, *b*] | 0 < *a* ≤ *b*}.

The C-OWA operator is not only bounded but also monotonic and associated with both the argument values and *Q* [28].

Subsequently, Yager and Xu [32] proposed the continuous ordered weighted geometric (C-OWG) operator based on the C-OWA operator and the geometric mean.

**Definition 13 (see [32])**

A continuous ordered weighted geometric (C-OWG) operator is a mapping *P*_*Q*_^*G*^ : *Ω* ^+^ → *R* ^+^ which is defined as follows:

(14) $$\begin{array}{c} P_{Q}^{G}\left(\left[a,b\right]\right)=b{\left(\frac{a}{b}\right)}^{\int_{0}^{1}(dQ(y)/dy)y\;dy}, \end{array}$$

where *Q* is a BUM function *Q* : [0,1]→[0,1] and is monotonic with the properties (1)  *Q*(0) = 0, (2)  *Q*(1) = 1, and (3)  *Q*(*x*) ≥ *Q*(*y*) if *x* > *y*. *Ω* ^+^ = {[*a*, *b*] | 0 < *a* ≤ *b*}.

**Lemma 14 (see [40])**

Let *x*_*j*_ > 0, *λ*_*j*_ > 0, *j* = 1,2,…, *n*, and ∑_*j*=1_^*n*^ *λ*_*j*_ = 1; then

(15) $$\begin{array}{c} \overset{n}{\underset{j=1}{\sum }}\lambda_{j}x_{j}\geq \overset{n}{\underset{j=1}{\prod }}{\left(x_{j}\right)}^{\lambda_{j}}. \end{array}$$

**Proposition 15**

For a closed interval [*a*, *b*], *Q* is a BUM function; then

(16) $$\begin{array}{c} P_{Q}^{A}\left(\left[a,b\right]\right)\geq P_{Q}^{G}\left(\left[a,b\right]\right). \end{array}$$

**Proof**

Since

(17) PQA([a,b])=∫01dQ(y)dy[b−(b−a)y]dy≈∑j=1n[Q(jΔy)−Q((j−1)Δy)]Δy  ×[b−(b−a)Δy],PQG([a,b])=b(ab)∫01(dQ(y)/dy)y dy ≈∏j=1n(b(ab)Δy)[Q(jΔy)−Q((j−1)Δy)]/Δy,

according to Lemma 14, we have

(18) ∑j=1n[Q(jΔy)−Q((j−1)Δy)]Δy[b−(b−a)Δy]  ≥∏j=1n(b(ab)Δy)[Q(jΔy)−Q((j−1)Δy)]/Δy,

thus

(19) $$\begin{array}{c} P_{Q}^{A}\left(\left[a,b\right]\right)\geq P_{Q}^{G}\left(\left[a,b\right]\right). \end{array}$$

## 3. Continuous Hesitant Fuzzy Ordered Weighted Aggregation Operators

In this section, some novel continuous ordered weighted aggregation operators are proposed to aggregate an IVHFE, such as the continuous hesitant fuzzy ordered weighted averaging (C-HFOWA) operator and the continuous hesitant fuzzy ordered weighted geometric (C-HFOWG) operator. Some essential properties of these operators are also studied in detail.

### 3.1. Continuous Hesitant Fuzzy Ordered Weighted Averaging Operator

**Definition 16**

A continuous HFOWA (C-HFOWA) operator is a mapping $f_{Q}^{A}:\tilde{H}\to H$, which has associated with it a BUM function *Q* : [0,1]→[0,1] having the properties (1) *Q*(0) = 0, (2) *Q*(1) = 1, and (3)  *Q*(*x*) ≥ *Q*(*y*) if *x* > *y*, such that

(20) fQA(h~)=⋃γ~∈h~{1−exp⁡(∫01dQ(x)dxln⁡⁡[1−γ~U+(γ~U−γ~L)x]dx)}.

The motivation behind the above definition is as follows. In fact, since $[{\tilde{\gamma }}^{L},{\tilde{\gamma }}^{U}]\in \tilde{h}$ is an interval whose arguments are preordered thus we do not need a reordering step, *Q*(*x*) is a BUM function, and *w*_*j*_ = *Q*(*j*/*n*) − *Q*(*j* − 1/*n*) (*j* = 1,2,…, *n*), which satisfy the conditions *w*_*j*_ ≥ 0 and ∑_*j*=1_^*n*^ *w*_*j*_ = 1. Based on Definition 5, (3), we have

(21) fQA(h~)≈⋃γ~∈h~{1−∏j=1n[1−(γσ(j)U −jn(γσ(j)U−γσ(j)L))][Q(j/n)−Q((j−1)/n)]}.

Let Δ*x* = 1/*n*; we get

(22) fQA(h~)≈⋃γ~∈h~{1−∏j=1n[1−(γσ(j)U−jΔx(γσ(j)U−γσ(j)L))][Q(jΔx)−Q((j−1)Δx)]}=⋃γ~∈h~{1−exp⁡(∑j=1n[Q(jΔx)−Q((j−1)Δx)]Δx ×ln⁡[1−(γσ(j)U−jΔx ×(γσ(j)U−γσ(j)L))]Δx)}.

When *n* → +*∞*, denote *x* = *j*Δ*x*, and *j* ranges from 0 to *n*, then we have *x* ∈ [0,1], and thus $f_{Q}^{A}(\tilde{h})={⋃}_{\tilde{\gamma }\in \tilde{h}}\left\{1-\exp\left(\int_{0}^{1}(dQ(x)/dx)\ln[1-{\tilde{\gamma }}^{U}+({\tilde{\gamma }}^{U}-{\tilde{\gamma }}^{L})x]dx\right)\right\}$.

From Definition 16 and the above analysis, we know that the aggregated result of the C-HFOWA operator is a HFE and the number of its possible membership values is the same as the one of the IVHFE to be aggregated; that is, $\#f_{Q}^{A}(\tilde{h})=\#\tilde{h}$.

**Example 17**

Let $\tilde{h}=\{[0.3,0.5],[0.5,0.8],[0.7,0.9]\}$ be an IVHFE, and *Q*(*x*) = *x* ^2^; then

(23) fQA(h~) =⋃γ~∈h~{1−exp⁡⁡(∫012xln⁡[1−0.5+(0.5−0.3)x]dx),   1−exp⁡(∫012xln⁡[1−0.8+(0.8−0.5)x]dx),  1−exp⁡(∫012xln⁡[1−0.9+(0.9−0.7)x]dx)} ={0.37,0.61,0.77}.

The C-HFOWA operator has the following essential properties.

**Proposition 18 (Bounded)**

For an IVHFE $\tilde{h}={⋃}_{\tilde{\gamma }\in \tilde{h}}\left\{\tilde{\gamma }=[{\tilde{\gamma }}^{L},{\tilde{\gamma }}^{U}]\right\}$, then

(24) $$\begin{array}{c} {⋃}_{\tilde{\gamma }\in \tilde{h}}\left\{{\tilde{\gamma }}^{L}\right\}\leq f_{Q}^{A}\left(\tilde{h}\right)\leq {⋃}_{\tilde{\gamma }\in \tilde{h}}\left\{{\tilde{\gamma }}^{U}\right\}. \end{array}$$

**Proof**

For any $\tilde{\gamma }=[{\tilde{\gamma }}^{L},{\tilde{\gamma }}^{U}]\in \tilde{h}$, when 0 ≤ *x* ≤ 1, we have $\ln(1-{\tilde{\gamma }}^{U})\leq \ln[1-{\tilde{\gamma }}^{U}+({\tilde{\gamma }}^{U}-{\tilde{\gamma }}^{L})x]\leq \ln(1-{\tilde{\gamma }}^{L})$. Since *Q*(*x*) ≥ *Q*(*y*) if *x* ≥ *y*, then *dQ*(*x*)/*dx* ≥ 0, we have $\ln(1-{\tilde{\gamma }}^{U})\int_{0}^{1}\left(dQ(x)/dx\right)dx\leq \int_{0}^{1}\left(dQ(x)/dx\right)\ln\left[1-{\tilde{\gamma }}^{U}+\left({\tilde{\gamma }}^{U}-{\tilde{\gamma }}^{L}\right)x\right]dx\leq \ln(1-{\tilde{\gamma }}^{L})\int_{0}^{1}\left(dQ(x)/dx\right)dx.\;$ According to ∫_0_^1^(*dQ*(*x*)/*dx*)*dx* = *Q*(1) − *Q*(0) = 1, we can obtain

(25) ln⁡(1−γ~U)≤∫01dQ(x)dxln⁡⁡[1−γ~U+(γ~U−γ~L)x]dx≤ln⁡⁡(1−γ~L)⟺1−γ~U≤exp⁡(∫01dQ(x)dx⁡×ln⁡[1−γ~U+(γ~U−γ~L)x]dx)≤1−γ~L⟺γ~L≤1−exp⁡(∫01dQ(x)dx⁡×ln⁡[1−γ~U+(γ~U−γ~L)x]dx)≤γ~U.

According to the extension principle of HFS, we have

(26) ⋃γ~∈h~{γ~L}≤⋃γ~∈h~{1−exp⁡(∫01dQ(x)dx ×ln⁡[1−γ~U+(γ~U−γ~L)x]dx)}≤⋃γ~∈h~{γ~U},

thus ${⋃}_{\tilde{\gamma }\in \tilde{h}}\left\{{\tilde{\gamma }}^{L}\right\}\leq f_{Q}(\tilde{h})\leq {⋃}_{\tilde{\gamma }\in \tilde{h}}\left\{{\tilde{\gamma }}^{U}\right\}$.

**Proposition 19 (Idempotency)**

For an IVHFE $\tilde{h}={⋃}_{\tilde{\gamma }\in \tilde{h}}\left\{\tilde{\gamma }=[{\tilde{\gamma }}^{L},{\tilde{\gamma }}^{U}]\right\}$, if all ${\tilde{\gamma }}^{L}={\tilde{\gamma }}^{U}$, then $\tilde{h}$ is reduced to a HFE *h* = ⋃_*γ*∈*h*_{*γ*}, and thus $f_{Q}^{A}(\tilde{h})=h$.

**Proof**

Consider

(27) fQA(h~)  =⋃γ~∈h~{1−exp⁡(∫01dQ(x)dxln⁡⁡[1−γ~U+(γ~U−γ~L)x]dx)}  =⋃γ~∈h~{1−exp⁡(∫01dQ(x)dxln⁡⁡[1−γ~U]dx)}=⋃γ~∈h~{1−exp⁡⁡(ln⁡⁡[1−γ~U]∫01dQ(x)dxdx)}  =⋃γ~∈h~{1−exp⁡⁡(ln⁡⁡[1−γ~U])}=⋃γ~∈h~{γ~U}=h.

**Proposition 20 (Monotonicity for $\tilde{h}$)**

For any two IVHFEs $\tilde{h}={⋃}_{\tilde{\gamma }\in \tilde{h}}\left\{\tilde{\gamma }=[{\tilde{\gamma }}^{L},{\tilde{\gamma }}^{U}]\right\}$ and ${\tilde{h}}^{\prime }={⋃}_{{\tilde{\gamma }}^{\prime }\in {\tilde{h}}^{\prime }}\left\{{\tilde{\gamma }}^{\prime }=[{\tilde{\gamma }}^{\prime L},{\tilde{\gamma }}^{\prime U}]\right\}$, if $\tilde{\gamma }\leq {\tilde{\gamma }}^{\prime }$for all $\tilde{\gamma }\in \tilde{h}$,  ${\tilde{\gamma }}^{\prime }\in {\tilde{h}}^{\prime }$, then $f_{Q}^{A}(\tilde{h})\leq f_{Q}^{A}({\tilde{h}}^{\prime })$.

**Proof**

Since $\tilde{\gamma }=[{\tilde{\gamma }}^{L},{\tilde{\gamma }}^{U}]\leq {\tilde{\gamma }}^{\prime }=[{\tilde{\gamma }}^{\prime L},{\tilde{\gamma }}^{\prime U}]$, we have

(28) (γ~′U−γ~U)  ≥(γ~′U−γ~U)(1−x), (x∈[0,1])  ⟺1−γ~U+(γ~U−γ~L)x≥1−γ~′U+(γ~′U−γ~′L)x  ⟺exp⁡(∫01dQ(x)dxln⁡⁡[1−γ~U+(γ~U−γ~L)x]dx)  ≥exp⁡⁡(∫01dQ(x)dxln⁡⁡[1−γ~′U+(γ~′U−γ~′L)x]dx)  ⟺1−exp⁡(∫01dQ(x)dxln⁡⁡[1−γ~U   +(γ~U−γ~L)x]dx)  ≤1−exp⁡(∫01dQ(x)dxln⁡⁡[1−γ~′U  +(γ~′U−γ~′L)x]dx).

According to the extension principle of HFS, we have

(29) ⋃γ~∈h~{1−exp⁡⁡(∫01dQ(x)dxln⁡⁡[1−γ~U+(γ~U−γ~L)x]dx)} ≤⋃γ~′∈h~′{1−exp⁡⁡(∫01dQ(x)dxln⁡⁡[1−γ~′U+(γ~′U−γ~′L)  ×x]dx)},

thus $f_{Q}(\tilde{h})\leq f_{Q}({\tilde{h}}^{\prime })$.

**Proposition 21 (Monotonicity for *Q*)**

For an IVHFE $\tilde{h}={⋃}_{\tilde{\gamma }\in \tilde{h}}\left\{\tilde{\gamma }=[{\tilde{\gamma }}^{L},{\tilde{\gamma }}^{U}]\right\}$, and *Q*_1_(*x*) ≥ *Q*_2_(*x*) for all *x* ∈ [0,1], then $f_{Q_{1}}^{A}(\tilde{h})\geq f_{Q_{2}}^{A}(\tilde{h})$.

**Proof**

Since

(30) fQ1A(h~)=⋃γ~∈h~{1−exp⁡(∫01dQ1(x)dx ×ln⁡[1−γ~U+(γ~U−γ~L)x]dx)},fQ2A(h~)=⋃γ~∈h~{1−exp⁡(∫01dQ2(x)dx ×ln⁡[1−γ~U+(γ~U−γ~L)x]dx)}

and $\ln[1-{\tilde{\gamma }}^{U}+({\tilde{\gamma }}^{U}-{\tilde{\gamma }}^{L})x]\leq 0$ for all *x* ∈ [0,1], so when 1 ≥ *Q*_1_(*x*) ≥ *Q*_2_(*x*) ≥ 0 for all *x* ∈ [0,1], we have

(31) ∫01dQ1(x)dxln⁡[1−γ~U+(γ~U−γ~L)x]dx  ≤∫01dQ2(x)dxln⁡[1−γ~U+(γ~U−γ~L)x]dx;

furthermore, the relation

(32) 1−exp⁡⁡(∫01dQ1(x)dxln⁡⁡[1−γ~U+(γ~U−γ~L)x]dx) ≥1−exp⁡⁡(∫01dQ2(x)dxln⁡⁡[1−γ~U+(γ~U−γ~L)x]dx)

holds, and thus we can get

(33) ⋃γ~∈h~{1−exp⁡⁡(∫01dQ1(x)dxln⁡⁡[1−γ~U+(γ~U−γ~L)x]dx)}  ≥⋃γ~∈h~{1−exp⁡(∫01dQ2(x)dxln⁡⁡[1−γ~U  +(γ~U−γ~L)x]dx)};

that is, $f_{Q_{1}}^{A}(\tilde{h})\geq f_{Q_{2}}^{A}(\tilde{h})$.

**Proposition 22**

For an IVHFE $\tilde{h}={⋃}_{\tilde{\gamma }\in \tilde{h}}\left\{\tilde{\gamma }=[{\tilde{\gamma }}^{L},{\tilde{\gamma }}^{U}]\right\}$, and *λ* > 0, then,

(34) $$\begin{array}{c} f_{Q}^{A}\left(\lambda \tilde{h}\right)=\lambda f_{Q}^{A}\left(\tilde{h}\right). \end{array}$$

**Proof**

Since

(35) λfQA(h~)=⋃γ~∈h~{1−(1−1+exp⁡(∫01dQ(x)dxln⁡[1−γ~U +(γ~U−γ~L)x]dx))λ}=⋃γ~∈h~{1−(exp⁡(∫01dQ(x)dxln⁡[1−γ~U +(γ~U−γ~L)x]dx))λ},fQA(λh~)≈⋃γ~∈h~{1−∏j=1n[1−(γσ(j)U−jΔx(γσ(j)U  −γσ(j)L))]λ[Q(jΔx)−Q((j−1)Δx)]}=⋃γ~∈h~{1−exp⁡(λ∑j=1n[Q(jΔx)−Q((j−1)Δx)]Δx×ln⁡⁡[1−(γσ(j)U−jΔx ×(γσ(j)U−γσ(j)L))]Δx)}.

When *n* → +*∞*, denote *x* = *j*Δ*x*, and *j* ranges from 0 to *n*, then we have *x* ∈ [0,1], and then $f_{Q}^{A}\left(\lambda \tilde{h}\right)={⋃}_{\tilde{\gamma }\in \tilde{h}}\{1-\exp(\lambda \int_{0}^{1}(dQ(x)/dx)\ln[1-{\tilde{\gamma }}^{U}+\left({\tilde{\gamma }}^{U}-{\tilde{\gamma }}^{L}\right)x]dx)\}$. Thus $f_{Q}^{A}(\lambda \tilde{h})=\lambda f_{Q}^{A}(\tilde{h})$.

### 3.2. Continuous Hesitant Fuzzy Ordered Weighted Geometric Operator

**Definition 23**

A continuous HFOWG (C-HFOWG) operator is a mapping $f_{Q}^{G}:\tilde{H}\to H$, which has associated with it a BUM function: *Q* : [0,1]→[0,1] having the properties (1)  *Q*(0) = 0, (2)  *Q*(1) = 1, and (3) *Q*(*x*) ≥ *Q*(*y*) if *x* > *y*, such that

(36) $$\begin{array}{c} f_{Q}^{G}\left(\tilde{h}\right)={⋃}_{\tilde{\gamma }\in \tilde{h}}\left\{\exp\left(\overset{1}{\underset{0}{\int }}\frac{dQ\left(x\right)}{dx}\ln\left[{\tilde{\gamma }}^{U}{\left(\frac{{\tilde{\gamma }}^{L}}{{\tilde{\gamma }}^{U}}\right)}^{x}\right]dx\right)\right\}. \end{array}$$

Now let us investigate how we can obtain Definition 23. In fact, since $[{\tilde{\gamma }}^{L},{\tilde{\gamma }}^{U}]\in \tilde{h}$ is an interval whose arguments are preordered thus we do not need a reordering step; *Q*(*x*) is a BUM function and *w*_*j*_ = *Q*(*j*/*n*) − *Q*(*j* − 1/*n*)  (*j* = 1,2,…, *n*), which satisfy the conditions *w*_*j*_ ≥ 0 and ∑_*j*=1_^*n*^ *w*_*j*_ = 1. Based on Definition 5, (4), we have

(37) fQG(h~) ≈⋃γ~∈h~{∏j=1n[γσ(j)U×(γσ(j)Lγσ(j)U)j/n][Q(j/n)−Q((j−1)/n)]}.

Let Δ*x* = 1/*n*; we get

(38) fQG(h~) ≈⋃γ~∈h~{∏j=1n[γσ(j)U×(γσ(j)Lγσ(j)U)jΔx][Q(jΔx)−Q((j−1)Δx)]} =⋃γ~∈h~{exp⁡(∑j=1n[Q(jΔx)−Q((j−1)Δx)]Δx ×ln⁡⁡[γσ(j)U×(γσ(j)Lγσ(j)U)jΔx]Δx)}.

When *n* → +*∞*, denote *x* = *j*Δ*x*, and *j* ranges from 0 to *n*, then we have *x* ∈ [0,1], and thus $f_{Q}^{G}(\tilde{h})={⋃}_{\tilde{\gamma }\in \tilde{h}}\left\{\exp(\int_{0}^{1}(dQ(x)/dx)\ln[{\tilde{\gamma }}^{U}{\left({\tilde{\gamma }}^{L}/{\tilde{\gamma }}^{U}\right)}^{x}]dx)\right\}.$

From Definition 23 and the above analysis, we know that the aggregated result of the C-HFOWG operator is a HFE and the number of its possible membership values is the same as the one of the IVHFE to be aggregated; that is, $\#f_{Q}^{G}(\tilde{h})=\#\tilde{h}$.

**Example 24**

Let $\tilde{h}=\{[0.3,0.5],[0.5,0.8],[0.7,0.9]\}$ be an IVHE and *Q*(*x*) = *x* ^1/2^, *Q*(*x*) = *x*, and *Q*(*x*) = *x* ^2^, then

(39) fQG(h~)=⋃γ~∈h~{exp⁡⁡(∫01dQ(x)dxln⁡⁡[0.5(0.30.5)x]dx),  exp⁡⁡(∫01dQ(x)dxln⁡⁡[0.8(0.50.8)x]⁡dx),  exp⁡⁡(∫01dQ(x)dxln⁡⁡[0.9(0.70.9)x]⁡dx)},fQ=x1/2G(h~)=⋃γ~∈h~{exp⁡⁡(∫010.5x−0.5ln⁡⁡[0.5(0.30.5)x]dx),  exp⁡⁡(∫010.5x−0.5ln⁡⁡[0.8(0.50.8)x]dx),  exp⁡⁡(∫010.5x−0.5ln⁡[0.9(0.70.9)x]dx)}={0.42,0.68,0.83},fQ=xG(h~)=⋃γ~∈h~{exp⁡⁡(∫01ln⁡⁡[0.5(0.30.5)x]dx),  exp⁡⁡(∫01ln⁡[0.8(0.50.8)x]dx),  exp⁡⁡(∫01ln⁡⁡[0.9(0.70.9)x]dx)}={0.39,0.63,0.79},fQ=x2G(h~)=⋃γ~∈h~{exp⁡⁡(∫012xln⁡[0.5(0.30.5)x]dx),  exp⁡⁡(∫012xln⁡[0.8(0.50.8)x]dx),  exp⁡⁡(∫012xln⁡[0.9(0.70.9)x]dx)}={0.36,0.58,0.76}.

From Example 24, we can see that the aggregated results are different when different BUM functions are adopted in the example, which indicates that the C-HFOWG operator can reflect the decision maker's risk preferences by using different BUM functions. Moreover, the aggregated results derived by the C-HFOWG operator become smaller as the BUM function values decrease.

Similar to the C-HFOWA operator, the C-HFOWG operator has the following essential properties.

**Proposition 25 (Bounded)**

For an IVHFE $\tilde{h}={⋃}_{\tilde{\gamma }\in \tilde{h}}\left\{\tilde{\gamma }=[{\tilde{\gamma }}^{L},{\tilde{\gamma }}^{U}]\right\}$, then

(40) $$\begin{array}{c} {⋃}_{\tilde{\gamma }\in \tilde{h}}\left\{{\tilde{\gamma }}^{L}\right\}\leq f_{Q}^{G}\left(\tilde{h}\right)\leq {⋃}_{\tilde{\gamma }\in \tilde{h}}\left\{{\tilde{\gamma }}^{U}\right\}. \end{array}$$

**Proof**

For any $\tilde{\gamma }=[{\tilde{\gamma }}^{L},{\tilde{\gamma }}^{U}]\in \tilde{h}$, when 0 ≤ *x* ≤ 1, we have $\ln({\tilde{\gamma }}^{L})\leq \ln[{\tilde{\gamma }}^{U}{\left({\tilde{\gamma }}^{L}/{\tilde{\gamma }}^{U}\right)}^{x}]\leq \ln({\tilde{\gamma }}^{U})$. Since *Q*(*x*) ≥ *Q*(*y*) if *x* ≥ *y*, then *dQ*(*x*)/*dx* ≥ 0, we have$\;\ln\left({\tilde{\gamma }}^{L}\right)\int_{0}^{1}\left(dQ(x)/dx\right)dx\leq \int_{0}^{1}\left(dQ(x)/dx\right)\ln\left[{\tilde{\gamma }}^{U}{\left({\tilde{\gamma }}^{L}/{\tilde{\gamma }}^{U}\right)}^{x}\right]dx\leq \ln\left({\tilde{\gamma }}^{U}\right)\int_{0}^{1}\left(dQ(x)/dx\right)dx.$ According to ∫_0_^1^(*dQ*(*x*)/*dx*)*dx* = *Q*(1) − *Q*(0) = 1, we can obtain $\ln\left({\tilde{\gamma }}^{L}\right)\leq \int_{0}^{1}\left(dQ(x)/dx\right)\ln\left[{\tilde{\gamma }}^{U}{\left({\tilde{\gamma }}^{L}/{\tilde{\gamma }}^{U}\right)}^{x}\right]dx\leq \ln\left({\tilde{\gamma }}^{U}\right);\;$ furthermore,

(41) exp⁡⁡(ln⁡⁡(γ~L))≤exp⁡(∫01dQ(x)dxln⁡⁡[γ~U(γ~Lγ~U)x]dx)≤exp⁡(ln⁡(γ~U))⟺γ~L≤exp⁡(∫01dQ(x)dxln⁡⁡[γ~U(γ~Lγ~U)x]dx)≤γ~U.

According to the extension principle of HFS, we have

(42) ⋃γ~∈h~{γ~L}≤⋃γ~∈h~{exp⁡⁡(∫01dQ(x)dxln⁡⁡[γ~U(γ~Lγ~U)x]dx)}≤⋃γ~∈h~{γ~U};

thus ${⋃}_{\tilde{\gamma }\in \tilde{h}}\left\{{\tilde{\gamma }}^{L}\right\}\leq f_{Q}^{G}(\tilde{h})\leq {⋃}_{\tilde{\gamma }\in \tilde{h}}\left\{{\tilde{\gamma }}^{U}\right\}$.

**Proposition 26 (Idempotency)**

For an IVHFE   $\tilde{h}={⋃}_{\tilde{\gamma }\in \tilde{h}}\left\{\tilde{\gamma }=[{\tilde{\gamma }}^{L},{\tilde{\gamma }}^{U}]\right\}$, if all ${\tilde{\gamma }}^{L}={\tilde{\gamma }}^{U}$, then $\tilde{h}$ is reduced to a hesitant set *h* = ⋃_*γ*∈*h*_{*γ*}, and thus $f_{Q}^{G}(\tilde{h})=h$.

**Proof**

Consider

(43) fQG(h~)=⋃γ~∈h~{exp⁡⁡(∫01dQ(x)dxln⁡⁡[γ~U(γ~Uγ~L)x]dx)}=⋃γ~∈h~{exp⁡⁡(ln⁡⁡[γ~U]∫01dQ(x)dxdx)}=⋃γ~∈h~{γ~U}=h.

**Proposition 27 (Monotonicity for $\tilde{h}$)**

For any two IVHFEs $\tilde{h}={⋃}_{\tilde{\gamma }\in \tilde{h}}\left\{\tilde{\gamma }=[{\tilde{\gamma }}^{L},{\tilde{\gamma }}^{U}]\right\}$ and ${\tilde{h}}^{\prime }={⋃}_{{\tilde{\gamma }}^{\prime }\in {\tilde{h}}^{\prime }}\left\{{\tilde{\gamma }}^{\prime }=[{\tilde{\gamma }}^{\prime L},{\tilde{\gamma }}^{\prime U}]\right\}$, if $\tilde{\gamma }\leq {\tilde{\gamma }}^{\prime }$ for all $\tilde{\gamma }\in \tilde{h}$, ${\tilde{\gamma }}^{\prime }\in {\tilde{h}}^{\prime }$, then $f_{Q}^{G}(\tilde{h})\leq f_{Q}^{G}({\tilde{h}}^{\prime })$.

**Proof**

Since $\tilde{\gamma }=[{\tilde{\gamma }}^{L},{\tilde{\gamma }}^{U}]\leq {\tilde{\gamma }}^{\prime }=[{\tilde{\gamma }}^{\prime L},{\tilde{\gamma }}^{\prime U}]$, we have

(44) (γ~′U/γ~U)  ≥(γ~′Uγ~U)(1−x), (x∈[0,1])  ⟺γ~U(γ~Lγ~U)x≤γ~′U(γ~′Lγ~′U)x, (x∈[0,1])  ⟺exp⁡(ln⁡[γ~U(γ~Lγ~U)x])  ≤exp⁡(ln⁡[γ~′U(γ~′Lγ~′U)x]), (x∈[0,1])  ⟺exp⁡(∫01dQdxln⁡⁡[γ~U(γ~Lγ~U)x]dx)⁡  ≤exp⁡(∫01dQdxln⁡[γ~′U(γ~′Lγ~′U)x]dx)⁡.

According to the extension principle of HFS, we have

(45) ⋃γ~∈h~{exp⁡(∫01dQ(x)dxln⁡⁡[γ~U(γ~Lγ~U)x]dx)⁡}  ≤⋃γ~′∈h~′{exp⁡⁡(∫01dQ(x)dxln⁡⁡[γ~′U(γ~′Lγ~′U)x]dx)};

thus, $f_{Q}^{G}\left(\tilde{h}\right)\leq f_{Q}^{G}\left({\tilde{h}}^{\prime }\right)$.

**Proposition 28 (Monotonicity for *Q*)**

For an IVHFE $\tilde{h}={⋃}_{\tilde{\gamma }\in \tilde{h}}\left\{\tilde{\gamma }=[{\tilde{\gamma }}^{L},{\tilde{\gamma }}^{U}]\right\}$, and *Q*_1_(*x*) ≥ *Q*_2_(*x*) for all *x* ∈ [0,1], then $f_{Q_{1}}^{G}(\tilde{h})\geq f_{Q_{2}}^{G}(\tilde{h})$.

**Proof**

Since

(46) $$\begin{array}{c} f_{Q_{1}}^{G}\left(\tilde{h}\right)={⋃}_{\tilde{\gamma }\in \tilde{h}}\left\{\exp\left(\overset{1}{\underset{0}{\int }}\frac{dQ_{1}\left(x\right)}{dx}\ln\left[{\tilde{\gamma }}^{U}{\left(\frac{{\tilde{\gamma }}^{U}}{{\tilde{\gamma }}^{L}}\right)}^{x}\right]dx\right)\right\},\\ f_{Q_{2}}^{G}\left(\tilde{h}\right)={⋃}_{\tilde{\gamma }\in \tilde{h}}\left\{\exp\left(\overset{1}{\underset{0}{\int }}\frac{dQ_{2}\left(x\right)}{dx}\ln\left[{\tilde{\gamma }}^{U}{\left(\frac{{\tilde{\gamma }}^{U}}{{\tilde{\gamma }}^{L}}\right)}^{x}\right]dx\right)\right\}, \end{array}$$

and $\ln[{\tilde{\gamma }}^{U}{\left({\tilde{\gamma }}^{L}/{\tilde{\gamma }}^{U}\right)}^{x}]\leq 0$ for all *x* ∈ [0,1], when 1 ≥ *Q*_1_(*x*) ≥ *Q*_2_(*x*) ≥ 0 for all *x* ∈ [0,1], we have $\int_{0}^{1}\left(dQ_{1}(x)/dx\right)\ln[{\tilde{\gamma }}^{U}{\left({\tilde{\gamma }}^{L}/{\tilde{\gamma }}^{U}\right)}^{x}]dx\leq \int_{0}^{1}\left(dQ_{2}(x)/dx\right)\ln[{\tilde{\gamma }}^{U}{\left({\tilde{\gamma }}^{L}/{\tilde{\gamma }}^{U}\right)}^{x}]dx;$ furthermore, the relation

(47) 1−exp⁡⁡(∫01dQ1(x)dxln⁡⁡[γ~U(γ~Lγ~U)x]dx)  ≥1−exp⁡⁡(∫01dQ2(x)dxln⁡⁡[γ~U(γ~Lγ~U)x]dx)

holds, and thus we can get

(48) ⋃γ~∈h~{1−exp⁡(∫01dQ1(x)dxln⁡[γ~U(γ~Lγ~U)x]dx)}  ≥⋃γ~∈h~{1−exp⁡⁡(∫01dQ2(x)dxln⁡⁡[γ~U(γ~Lγ~U)x]dx)},

that means $f_{Q_{1}}^{G}(\tilde{h})\geq f_{Q_{2}}^{G}(\tilde{h})$.

**Proposition 29**

For an IVHFE $\tilde{h}={⋃}_{\tilde{\gamma }\in \tilde{h}}\left\{\tilde{\gamma }=[{\tilde{\gamma }}^{L},{\tilde{\gamma }}^{U}]\right\}$, and *λ* > 0, then

(49) $$\begin{array}{c} f_{Q}^{G}\left({\tilde{h}}^{\lambda }\right)={\left(f_{Q}^{G}\left(\tilde{h}\right)\right)}^{\lambda }. \end{array}$$

**Proof**

Since

(50) (fQG(h~))λ =⋃γ~∈h~{(exp⁡⁡(∫01dQ(x)dxln⁡⁡[γ~U(γ~Lγ~U)x]dx))λ} =⋃γ~∈h~{exp⁡⁡(λ∫01dQ(x)dxln⁡⁡[γ~U(γ~Lγ~U)x]dx)},fQG(h~λ)  =⋃γ~∈h~{exp⁡⁡(∫01λdQ(x)dxln⁡⁡[γ~U(γ~Lγ~U)x]dx)}  =⋃γ~∈h~{exp⁡(λ∫01dQ(x)dxln⁡[γ~U(γ~Lγ~U)x]dx)}.

Thus, $f_{Q}^{G}\left({\tilde{h}}^{\lambda }\right)={\left(f_{Q}^{G}\left(\tilde{h}\right)\right)}^{\lambda }$.

## 4. Extended C-HFOW Operators

In order to aggregate multiple IVHFEs, we extend the C-HFOW (C-HFOWA and C-HFOWG) operators to the case where the given inputs are multiple IVHFEs of dimension *n* and develop some extended C-HFOW operators.

### 4.1. Weighted C-HFOW Operators

**Definition 30**

Let ${\tilde{h}}_{j}\;(j=1,2,\ldots ,n)$ be a collection of IVHFEs, and let *ω* = (*ω*_1_, *ω*_2_,…, *ω*_*n*_) be the relative weight vector of ${\tilde{h}}_{j}\;(j=1,2,\ldots ,n)$, with *ω*_*i*_ ∈ [0,1] and ∑_*i*=1_^*n*^ *ω*_*i*_ = 1. A weighted C-HFOWA (WC-HFOWA) operator is a mapping $\text{WC-HFOWA}:{\tilde{H}}^{n}\to H$, according to the following expression:

(51) WC-HFOWA(h~1,h~2,…,h~n)=HFWA(fQA(h~1),fQA(h~2),…,fQA(h~n))=⋃fQA(γ~j)∈fQA(h~j),j=1,2,…,n{1−∏j=1n(1−fQA(γ~j))ωj}.

It is natural that the aggregated result derived from the WC-HFOWA operator is a HFE and the number of the possible aggregated values satisfies the following inequality:

(52) $$\begin{array}{c} 1\leq \#\text{WC-HFOWA}\left({\tilde{h}}_{j},j=1,2,\ldots ,n\right)\leq \overset{n}{\underset{j=1}{\prod }}\#{\tilde{h}}_{j}. \end{array}$$

Clearly, if all possible aggregated values in the derived HFE are identical, then $\#\text{WC-HFOWA}\;({\tilde{h}}_{j},j=1,2,\ldots ,n)=1$, on the contrary, if the all possible aggregated values in the derived HFE are different, then $\#\text{WC-HFOWA}\;({\tilde{h}}_{j},j=1,2,\ldots ,n)=\prod_{j=1}^{n}\#{\tilde{h}}_{j}$.

On the basis of the properties of the C-HFOWA operator, we can further obtain some properties of WC-HFOWA operator.

**Proposition 31 (Idempotency)**

Let ${\tilde{h}}_{j}={⋃}_{{\tilde{\gamma }}_{j}\in {\tilde{h}}_{j}}\left\{{\tilde{\gamma }}_{j}\right\}\;(j=1,2,\ldots ,n)$ be a collection of IVHFEs, if  ${\tilde{h}}_{j}=\tilde{h}\;(j=1,2,\ldots ,n)$, then

(53) $$\begin{array}{c} \text{WC-HFOWA}\left({\tilde{h}}_{1},{\tilde{h}}_{2},\ldots ,{\tilde{h}}_{n}\right)=f_{Q}^{A}\left(\tilde{h}\right). \end{array}$$

**Proof**

Since ${\tilde{h}}_{j}=\tilde{h}\;(j=1,2,\ldots ,n)$, we have

(54) WC-HFOWA(h~1,h~2,…,h~n) =⋃fQA(γ~j)∈fQA(h~j),j=1,2,…,n{1−∏j=1n(1−fQA(γ~j))ωj} =⋃fQA(γ~)∈fQA(h~),j=1,2,…,n{1−∏j=1n(1−fQA(γ~))ωj} =⋃fQA(γ~)∈fQA(h~){fQA(γ~)}=fQA(h~).

**Proposition 32 (Bounded 1)**

Let ${\tilde{h}}_{j}={⋃}_{{\tilde{\gamma }}_{j}\in {\tilde{h}}_{j}}\left\{{\tilde{\gamma }}_{j}\right\}\;(j=1,2,\ldots ,n)$ be a collection of IVHFEs, ${⋂}_{j=1}^{n}{\tilde{h}}_{j}^{L}={⋃}_{{\tilde{\gamma }}_{j}\in {\tilde{h}}_{j},j=1,2,\ldots ,n}\left\{\min_{1\leq j\leq n}\{{\tilde{\gamma }}_{j}^{L}\}\right\}$, and ${⋃}_{j=1}^{n}{\tilde{h}}_{j}^{U}={⋃}_{{\tilde{\gamma }}_{j}\in {\tilde{h}}_{j},j=1,2,\ldots ,n}\left\{\max_{1\leq j\leq n}\{{\tilde{\gamma }}_{j}^{U}\}\right\}$, then

(55) $$\begin{array}{c} \overset{n}{\underset{j=1}{⋂}}{\tilde{h}}_{j}^{L}\leq \text{ WC-HFOWA }\left({\tilde{h}}_{1},{\tilde{h}}_{2},\ldots ,{\tilde{h}}_{n}\right)\leq \overset{n}{\underset{j=1}{⋃}}{\tilde{h}}_{j}^{U}. \end{array}$$

**Proof**

Let ${⋃}_{{\tilde{\gamma }}_{j}\in {\tilde{h}}_{j},j=1,2,\ldots ,n}\left\{{\tilde{\gamma }}_{j}^{-}\right\}={⋃}_{{\tilde{\gamma }}_{j}\in {\tilde{h}}_{j},j=1,2,\ldots ,n}\left\{\min_{1\leq j\leq n}\{{\tilde{\gamma }}_{j}^{L}\}\right\}$ and ${⋃}_{{\tilde{\gamma }}_{j}\in {\tilde{h}}_{j},j=1,2,\ldots ,n}\left\{{\tilde{\gamma }}_{j}^{+}\right\}={⋃}_{{\tilde{\gamma }}_{j}\in {\tilde{h}}_{j},j=1,2,\ldots ,n}\left\{\max_{1\leq j\leq n}\{{\tilde{\gamma }}_{j}^{U}\}\right\}$. We have ${⋃}_{{\tilde{\gamma }}_{j}\in {\tilde{h}}_{j},j=1,2,\ldots ,n}\left\{{\tilde{\gamma }}_{j}^{-}\right\}={⋃}_{{\tilde{\gamma }}_{j}\in {\tilde{h}}_{j},j=1,2,\ldots ,n}\left\{\min_{1\leq j\leq n}\{{\tilde{\gamma }}_{j}^{L}\}\right\}\leq {⋃}_{f_{Q}^{A}({\tilde{\gamma }}_{j})\in f_{Q}^{A}({\tilde{h}}_{j}),j=1,2,\ldots ,n}\left\{1-{\prod_{j=1}^{n}\left(1-f_{Q}^{A}({\tilde{\gamma }}_{j})\right)}^{\omega_{j}}\right\}\leq {⋃}_{{\tilde{\gamma }}_{j}\in {\tilde{h}}_{j},j=1,2,\ldots ,n}\left\{\max_{1\leq j\leq n}\{{\tilde{\gamma }}_{j}^{U}\}\right\}={⋃}_{{\tilde{\gamma }}_{j}\in {\tilde{h}}_{j},j=1,2,\ldots ,n}\left\{{\tilde{\gamma }}_{j}^{+}\right\}$. Therefore, ${⋂}_{j=1}^{n}{\tilde{h}}_{j}^{L}\leq \text{WC-HFOWA}\;({\tilde{h}}_{1},{\tilde{h}}_{2},\ldots ,{\tilde{h}}_{n})\leq {⋃}_{j=1}^{n}{\tilde{h}}_{j}^{U}$.

**Proposition 33 (Bounded 2)**

Let ${\tilde{h}}_{j}={⋃}_{{\tilde{\gamma }}_{j}\in {\tilde{h}}_{j}}\left\{{\tilde{\gamma }}_{j}\right\}\;(j=1,2,\ldots ,n)$ be a collection of IVHFEs; then

(56) min⁡j{s(fQA(h~j))}≤s(
WC-HFOWA
(h~1,h~2,…,h~n))≤max⁡j{s(fQA(h~j))}.

**Proof**

Without loss of generality, assume that

(57) min⁡j{s(fQA(h~j))}=s(fQA(h~k))=1#h~k∑fQA(γ~k)∈fQA(h~k)fQA(γ~k),s(WC-HFOWA(h~1,h~2,…,h~n))=1∏j=1n#h~j∑fQA(γ~j)∈fQA(h~j),j=1,2,…,n(1−∏j=1n(1−fQA(γ~j))ωj)=1#h~k(1∏j=1,j≠kn#h~j×∑fQA(γ~j)∈fQA(h~j),j=1,2,…,n(1−∏j=1n(1−fQA(γ~j))ωj))≥1#h~k∑fQA(γ~k)∈fQA(h~k)fQA(γ~k)=min⁡j{s(fQA(h~j))}.

Similarly, we can get

(58) s(WC-HFOWA(h~1,h~2,…,h~n))=1#h~k(1∏j=1,j≠kn#h~j ×∑fQA(γ~j)∈fQA(h~j),j=1,2,…,n(1−∏j=1n(1−fQA(γ~j))ωj))≤max⁡j{s(fQA(h~j))}.

Thus $\min_{j}\{s(f_{Q}^{A}({\tilde{h}}_{j}))\}\leq s(\text{WC-HFOWA}({\tilde{h}}_{1},{\tilde{h}}_{2},\ldots ,{\tilde{h}}_{n}))\leq \max_{j}\{s(f_{Q}^{A}({\tilde{h}}_{j}))\}$.

**Proposition 34 (Monotonicity)**

Let ${\tilde{h}}_{j}={⋃}_{{\tilde{\gamma }}_{j}\in {\tilde{h}}_{j}}\left\{{\tilde{\gamma }}_{j}\right\}$ and ${\tilde{h}}_{j}^{\prime }={⋃}_{{\tilde{\gamma }}_{j}\in {\tilde{h}}_{j}}\left\{{\tilde{\gamma }}_{j}^{\prime }\right\}\;(j=1,2,\ldots ,n)$ be two collections of IVHFEs; If ${\tilde{\gamma }}_{j}\leq {\tilde{\gamma }}_{j}^{\prime }$ for all ${\tilde{\gamma }}_{j}\in {\tilde{h}}_{j}$, ${\tilde{\gamma }}_{j}^{\prime }\in {\tilde{h}}_{j}^{\prime }$, *j* = 1,2,…, *n*, then

(59) WC-HFOWA(h~1,h~2,…,h~n)  ≤WC-HFOWA(h~1′,h~2′,…,h~n′).

**Proof**

Since ${\tilde{\gamma }}_{j}\leq {\tilde{\gamma }}_{j}^{\prime }$ for all ${\tilde{\gamma }}_{j}\in {\tilde{h}}_{j}$, ${\tilde{\gamma }}_{j}^{\prime }\in {\tilde{h}}_{j}^{\prime }$, *j* = 1,2,…, *n*, we have $f_{Q}^{A}({\tilde{\gamma }}_{j})\leq f_{Q}^{A}({\tilde{\gamma }}_{j}^{\prime })$, and then

(60) ⋃fQA(γ~j)∈fQA(h~j),j=1,2,…,n{1−∏j=1n(1−fQA(γ~j))ωj}  ≤⋃fQA(γ~j′)∈fQA(h~j′),j=1,2,…,n{1−∏j=1n(1−fQA(γ~j′))ωj}.

Thus $\text{WC-HFOWA }\left({\tilde{h}}_{1},{\tilde{h}}_{2},\ldots ,{\tilde{h}}_{n}\right)\leq \text{WC-HFOWA}({\tilde{h}}_{1}^{\prime },{\tilde{h}}_{2}^{\prime },\ldots ,{\tilde{h}}_{n}^{\prime })$.

**Definition 35**

Let ${\tilde{h}}_{j}\;(j=1,2,\ldots ,n)$ be a collection of IVHFEs, let *ω* = (*ω*_1_, *ω*_2_,…, *ω*_*n*_) be the relative weight vector of ${\tilde{h}}_{j}\;(j=1,2,\ldots ,n)$, with *ω*_*i*_ ∈ [0,1], and ∑_*i*=1_^*n*^ *ω*_*i*_ = 1. A weighted C-HFOWG (WC-HFOWG) operator is a mapping $\text{WC-HFOWG}:{\tilde{H}}^{n}\to H$, according to the following expression:

(61) WC-HFOWG(h~1,h~2,…,h~n)  =HFWG(fQG(h~1),fQG(h~2),…,fQG(h~n))  =⋃fQG(γ~j)∈fQG(h~j),j=1,2,…,n{∏j=1n(fQG(γ~j))ωj}.

It is natural that the aggregated result derived from the WC-HFOWG operator is a HFE and the number of the possible aggregated values satisfies the following inequality:

(62) $$\begin{array}{c} 1\leq \#\text{WC-HFOWG}\left({\tilde{h}}_{j},j=1,2,\ldots ,n\right)\leq \overset{n}{\underset{j=1}{\prod }}\#{\tilde{h}}_{j}. \end{array}$$

Clearly, if all possible aggregated values in the derived HFE are the identical, then $\#\text{WC-HFOWG}\;({\tilde{h}}_{j},j=1,2,\ldots ,n)=1$, and on the contrary, if the all possible aggregated values in the derived HFE are different, then $\#\text{WC-HFOWG }({\tilde{h}}_{j},j=1,2,\ldots ,n)=\prod_{j=1}^{n}\#{\tilde{h}}_{j}$.

The WC-HFOWG operator has similar properties with the WC-HFOWA operator.

**Proposition 36 (Idempotency)**

Let ${\tilde{h}}_{j}={⋃}_{{\tilde{\gamma }}_{j}\in {\tilde{h}}_{j}}\left\{{\tilde{\gamma }}_{j}\right\}\;(j=1,2,\ldots ,n)$ be a collection of IVHFEs; if  ${\tilde{h}}_{j}=\tilde{h}\;(j=1,2,\ldots ,n)$, then

(63) $$\begin{array}{c} \text{WC-HFOWA}\;\left({\tilde{h}}_{1},{\tilde{h}}_{2},\ldots ,{\tilde{h}}_{n}\right)=f_{Q}^{G}\left(\tilde{h}\right). \end{array}$$

**Proposition 37 (Bounded 1)**

Let ${\tilde{h}}_{j}={⋃}_{{\tilde{\gamma }}_{j}\in {\tilde{h}}_{j}}\left\{{\tilde{\gamma }}_{j}\right\}\;(j=1,2,\ldots ,n)$ be a collection of IVHFEs,$\;{⋂}_{j=1}^{n}{\tilde{h}}_{j}^{L}={⋃}_{{\tilde{\gamma }}_{j}\in {\tilde{h}}_{j},j=1,2,\ldots ,n}\left\{\min_{1\leq j\leq n}\{{\tilde{\gamma }}_{j}^{L}\}\right\}$, and ${⋃}_{j=1}^{n}{\tilde{h}}_{j}^{U}={⋃}_{{\tilde{\gamma }}_{j}\in {\tilde{h}}_{j},j=1,2,\ldots ,n}\left\{\max_{1\leq j\leq n}\{{\tilde{\gamma }}_{j}^{U}\}\right\}$, then

(64) $$\begin{array}{c} \overset{n}{\underset{j=1}{⋂}}{\tilde{h}}_{j}^{L}\leq \text{WC-HFOWA}\left({\tilde{h}}_{1},{\tilde{h}}_{2},\ldots ,{\tilde{h}}_{n}\right)\leq \overset{n}{\underset{j=1}{⋃}}{\tilde{h}}_{j}^{U}. \end{array}$$

**Proposition 38 (Bounded 2)**

Let ${\tilde{h}}_{j}={⋃}_{{\tilde{\gamma }}_{j}\in {\tilde{h}}_{j}}\left\{{\tilde{\gamma }}_{j}\right\}\;(j=1,2,\ldots ,n)$ be a collection of IVHFEs; then

(65) min⁡j{s(fQG(h~j))}≤s(WC-HFOWA(h~1,h~2,…,h~n))≤max⁡j{s(fQG(h~j))}.

**Proposition 39 (Monotonicity)**

Let ${\tilde{h}}_{j}={⋃}_{{\tilde{\gamma }}_{j}\in {\tilde{h}}_{j}}\left\{{\tilde{\gamma }}_{j}\right\}$ and ${\tilde{h}}_{j}^{\prime }={⋃}_{{\tilde{\gamma }}_{j}\in {\tilde{h}}_{j}}\left\{{\tilde{\gamma }}_{j}^{\prime }\right\}\;(j=1,2,\ldots ,n)$ be two collections of IVHFEs; If ${\tilde{\gamma }}_{j}\leq {\tilde{\gamma }}_{j}^{\prime }$ for all ${\tilde{\gamma }}_{j}\in {\tilde{h}}_{j}$, ${\tilde{\gamma }}_{j}^{\prime }\in {\tilde{h}}_{j}^{\prime }$, *j* = 1,2,…, *n*, then

(66) WC-HFOWA(h~1,h~2,…,h~n)  ≤WC-HFOWA(h~1′,h~2′,…,h~n′).

### 4.2. Ordered Weighted C-HFOW Operators

**Definition 40**

Let ${\tilde{h}}_{j}\;(j=1,2,\ldots ,n)$ be a collection of IVHFEs, and an ordered weighted C-HFOWA (OWC-HFOWA) operator is a mapping $\text{OWC-HFOWA}:{\tilde{H}}^{n}\to H$, associated with a weight vector *w* = (*w*_1_, *w*_2_,…, *w*_*n*_), such that *w*_*i*_ ∈ [0,1] and ∑_*i*=1_^*n*^ *w*_*i*_ = 1, according to the following expressions:

(67) OWC-HFOWA(h~1,h~2,…,h~n) =HFOWA(fQA(h~1),fQA(h~2),…,fQA(h~n)) =⋃fQA(γ~σ(j))∈fQA(h~σ(j)),j=1,2,…,n{1−∏j=1n(1−fQA(γ~σ(j)))wj},

where *σ*(·): {1,2,…, *n*}→{1,2,…, *n*} is a permutation function such that $f_{Q}^{A}({\tilde{\gamma }}_{\sigma (j)})$ is the *σ*(*j*)th largest element of the collection of $f_{Q}^{A}\left({\tilde{\gamma }}_{j}\right)\;(j=1,2,\ldots ,n)$, or

(68) OWC-HFOWA(h~1,h~2,…,h~n)  =HFOWA(fQA(h~1),fQA(h~2),…,fQA(h~n))  =⋃fQA(γ~j)∈fQA(h~j),j=1,2,…,n{1−∏j=1n(1−fQA(γ~j))wρ(j)},

where *ρ*(·): {1,2,…, *n*}→{1,2,…, *n*} is a permutation function such that $f_{Q}^{A}({\tilde{\gamma }}_{j})$ is the *ρ*(*j*)th largest element of the collection of $f_{Q}^{A}\left({\tilde{\gamma }}_{j}\right)\;(j=1,2,\ldots ,n)$.

**Definition 41**

Let ${\tilde{h}}_{j}\;(j=1,2,\ldots ,n)$ be a collection of IVHFEs, and an ordered weighted C-HFOWG (OWC-HFOWG) operator is a mapping $\text{OWC-HFOWG}:{\tilde{H}}^{n}\to H$, associated with a weight vector *w* = (*w*_1_, *w*_2_,…, *w*_*n*_), such that *w*_*i*_ ∈ [0,1] and ∑_*i*=1_^*n*^ *w*_*i*_ = 1, according to the following expressions:

(69) OWC-HFOWG(h~1,h~2,…,h~n) =HFOWG(fQG(h~1),fQG(h~2),…,fQG(h~n)) =⋃fQG(γ~σ(j))∈fQG(h~σ(j)),j=1,2,…,n{∏j=1n(fQG(γ~σ(j)))wj},

where *σ*(·): {1,2,…, *n*}→{1,2,…, *n*} is a permutation function such that $f_{Q}^{G}({\tilde{\gamma }}_{\sigma (j)})$ is the *σ*(*j*)th largest element of the collection of $f_{Q}^{G}\left({\tilde{\gamma }}_{j}\right)\;(j=1,2,\ldots ,n)$, or

(70) OWC-HFOWG(h~1,h~2,…,h~n)  =HFOWG(fQG(h~1),fQG(h~2),…,fQG(h~n))  =⋃fQG(γ~j)∈fQG(h~j),j=1,2,…,n{∏j=1n(fQG(γ~j))wρ(j)},

where *ρ*(·): {1,2,…, *n*}→{1,2,…, *n*} is a permutation function such that $f_{Q}^{G}({\tilde{\gamma }}_{j})$ is the *ρ*(*j*)th largest element of the collection of $f_{Q}^{G}({\tilde{\gamma }}_{j})(j=1,2,\ldots ,n)$.

It is natural that the aggregated result derived from the OWC-HFOW (OWC-HFOWA or OWC-HFOWG) operators is a HFE and the number of the possible aggregated values satisfies the following inequality:

(71) $$\begin{array}{c} 1\leq \#\text{OWC-HFOW }\left({\tilde{h}}_{j},j=1,2,\ldots ,n\right)\leq \overset{n}{\underset{j=1}{\prod }}\#{\tilde{h}}_{j}. \end{array}$$

Clearly, if all possible aggregated values in the derived HFE are identical, then $\#\text{OWC-HFOW}\;({\tilde{h}}_{j},j=1,2,\ldots ,n)=1$; on the contrary, if the all possible aggregated values in the derived HFE are different, then $\#\text{OWC-HFOW}\;({\tilde{h}}_{j},j=1,2,\ldots ,n)=\prod_{j=1}^{n}\#{\tilde{h}}_{j}$.

The OWC-HFOW operators have similar properties with the WC-HFOW operators; they are idempotent, bounded, monotonic, and so forth, and the proofs of them are omitted here for saving space.

From the above definitions, we know that the WC-HFOW (WC-HFOWA or WC-HFOWG) operators focus solely on the weight of the individual argument variable itself and ignore the associated (position) weight with respect to the individual argument variable value. However, the OWC-HFOW (OWC-HFOWA or OWC-HFOWG) operators focus on the associated (position) weight with respect to the individual argument variable value and ignore the weight of the individual argument variable itself. To generalize the WC-HFOW operators and the OWC-HFOW operators, motivated by the idea of the weighted OWA operator [41], the hybrid weighted aggregation operator [42] and the synergetic weighted aggregation operator [27], in the following, we present two synergetic weighted C-HFOW (SWC-HFOWA and SWC-HFOWG) operators.

### 4.3. Synergetic Weighted C-HFOW Operators

**Definition 42**

Let ${\tilde{h}}_{j}\;(j=1,2,\ldots ,n)$ be a collection of IVHFEs, and let *ω* = (*ω*_1_, *ω*_2_,…, *ω*_*n*_) be the relative weight vector of ${\tilde{h}}_{j}\;(j=1,2,\ldots ,n)$, with *ω*_*i*_ ∈ [0,1] and ∑_*i*=1_^*n*^ *ω*_*i*_ = 1. A synergetic weighted C-HFOWA (SWC-IVHFOWA) operator is a mapping $\text{SWC-HFOWA}:{\tilde{H}}^{n}\to H$, associated with a weight vector *w* = (*w*_1_, *w*_2_,…, *w*_*n*_), such that *w*_*i*_ ∈ [0,1] and ∑_*i*=1_^*n*^ *w*_*i*_ = 1, according to the following expression:

(72) SWC-HFOWA(h~1,h~2,…,h~n)=HFSWA(fQA(h~1),fQA(h~2),…,fQA(h~n))=⋃fQA(γ~j)∈fQA(h~j),j=1,2,…,n{1−∏j=1n(1−fQA(γ~j))wρ(j)ωj/∑j=1nwρ(j)ωj},

where *ρ*(·): {1,2,…, *n*}→{1,2,…, *n*} is a permutation function such that $f_{Q}^{A}({\tilde{\gamma }}_{j})$ is the *ρ*(*j*)th largest element of the collection of $f_{Q}^{A}\left({\tilde{\gamma }}_{j}\right)\;(j=1,2,\ldots ,n)$.

Alternatively, according to the Proposition 11, we can get an equivalent expression.

(73) SWC-HFOWA(h~1,h~2,…,h~n)=HFSWA(fQA(h~1),fQA(h~2),…,fQA(h~n))=⋃fQA(γ~j)∈fQA(h~j),j=1,2,…,n{1−∏j=1n(1−fQA(γ~σ(j)))wjωσ↦(j)/∑j=1nwjωσ↦(j)},

where *σ*(·): {1,2,…, *n*}→{1,2,…, *n*} is a permutation function such that $f_{Q}^{A}({\tilde{\gamma }}_{\sigma (j)})$ is the *σ*(*j*)th largest element of the collection of $f_{Q}^{A}\left({\tilde{\gamma }}_{j}\right)\;(j=1,2,\ldots ,n)$, and *σ* ↦ (·) is a permutation function, which corresponds to *σ*(·), for the relative weights *ω*_*j*_  (*j* = 1,2,…, *n*).

**Definition 43**

Let ${\tilde{h}}_{j}\;(j=1,2,\ldots ,n)$ be a collection of IVHFEs, and let *ω* = (*ω*_1_, *ω*_2_,…, *ω*_*n*_) be the relative weight vector of the ${\tilde{h}}_{j}\;(j=1,2,\ldots ,n)$. A synergetic weighted C-HFOWG (SWC-HFOWG) operator is a mapping $\text{SWC-HFOWG}:{\tilde{H}}^{n}\to H$, associated with a weight vector *w* = (*w*_1_, *w*_2_,…, *w*_*n*_), such that *w*_*i*_ ∈ [0,1] and ∑_*i*=1_^*n*^ *w*_*i*_ = 1, according to the following expression:

(74) SWC-HFOWG(h~1,h~2,…,h~n)=HFSWG(fQG(h~1),fQG(h~2),…,fQG(h~n))=⋃fQG(γ~j)∈fQG(h~j),j=1,2,…,n{∏j=1n(fQG(γ~j))(wρ(j)ωj/∑j=1nwρ(j)ωj)},

where *ρ*(·): {1,2,…, *n*}→{1,2,…, *n*} is a permutation function such that $f_{Q}^{G}({\tilde{\gamma }}_{j})$ is the *ρ*(*j*)th largest element of the collection of $f_{Q}^{G}\left({\tilde{\gamma }}_{j}\right)\;(j=1,2,\ldots ,n)$, or

(75) SWC-HFOWG(h~1,h~2,…,h~n)=HFSWG(fQG(h~1),fQG(h~2),…,fQG(h~n))=⋃fQG(γ~j)∈fQG(h~j),j=1,2,…,n{∏j=1n(fQG(γ~σ(j)))wjωσ↦(j)/∑j=1nwjωσ↦(j)},

where *σ*(·): {1,2,…, *n*}→{1,2,…, *n*} is a permutation function such that $f_{Q}^{G}({\tilde{\gamma }}_{\sigma (j)})$ is the *σ*(*j*)th largest element of the collection of $f_{Q}^{G}\left({\tilde{\gamma }}_{j}\right)\;(j=1,2,\ldots ,n)$, and *σ* ↦ (·) is a permutation function, which corresponds to *σ*(·), for the relative weights *ω*_*j*_  (*j* = 1,2,…, *n*).

It is natural that the result derived from the SWC-HFOW operators (AWC-HFOWA or SWC-HFOWG) is a HFE and the number of the possible aggregated values satisfies the following inequality:

(76) $$\begin{array}{c} 1\leq \#\text{SWC-HFOW}\left({\tilde{h}}_{j},j=1,2,\ldots ,n\right)\leq \overset{n}{\underset{j=1}{\prod }}\#{\tilde{h}}_{j}. \end{array}$$

Clearly, if all possible aggregated values in the derived HFE are the identical, then $\#\text{SWC-HFOW}\;({\tilde{h}}_{j},j=1,2,\ldots ,n)=1$; on the contrary, if the all possible aggregated values in the derived HFE are different, then $\#\text{SWC-HFOW}({\tilde{h}}_{j},j=1,2,\ldots ,n)=\prod_{j=1}^{n}\#{\tilde{h}}_{j}$.

With regard to the SWC-HFOW operators, we have the following propositions.

**Proposition 44**

If the relative weight vector *ω* = (*ω*_1_, *ω*_2_,…, *ω*_*n*_) = (1/*n*, 1/*n*,…, 1/*n*), then the SWC-HFOW operators are reduced to the OWC-HFOW operators:

(77) SWC-HFOW(h~1,h~2,…,h~n) →ωj=1/n,j=1,...,nOWC-HFOW(h~1,h~2,…,h~n).

**Proposition 45**

If the associated weight vector *w* = (*w*_1_, *w*_2_,…, *w*_*n*_) = (1/*n*, 1/*n*,…, 1/*n*), then the SWC-HFOW operators are reduced to the WC-HFOW operators:

(78) SWC-HFOW(h~1,h~2,…,h~n) →wj=1/n,j=1,...,nWC-HFOW(h~1,h~2,…,h~n).

**Proposition 46**

If the relative weight vector *ω* = (*ω*_1_, *ω*_2_,…, *ω*_*n*_) = (1/*n*, 1/*n*,…, 1/*n*) and the associated weight vector *w* = (*w*_1_, *w*_2_,…, *w*_*n*_) = (1/*n*, 1/*n*,…, 1/*n*), then the SWC-HFOW operators are reduced to averaging C-HFOW (AC-HFOW) operator:

(79) SWC-HFOW(h~1,h~2,…,h~n)  →ωj=1/n,j=1,…,nAC-HFOW(h~1,h~2,…,h~n).

Concretely, consider the averaging C-HFOWA operator:

(80) AC-HFOWA(h~1,h~2,…,h~n)  =HFA(fQA(h~1),fQA(h~2),…,fQA(h~n))    =⋃fQA(γ~j)∈fQA(h~j),j=1,2,…,n{1−∏j=1n(1−fQA(γ~j))1/n}.

And consider the geometric C-HFOWG operator

(81) GC-HFOWG(h~1,h~2,…,h~n)  =HFG(fQG(h~1),fQG(h~2),…,fQG(h~n))  =⋃fQG(γ~j)∈fQG(h~j),j=1,2,…,n{∏j=1n(fQG(γ~j))1/n}.

The proofs of them are intuitional and omitted here.

**Example 47**

Let ${\tilde{h}}_{1}=\{[0.5,0.7],[0.8,0.9]\}$, ${\tilde{h}}_{2}=\{[0.3,0.4],[0.4,0.5],[0.5,0.6]\}$, ${\tilde{h}}_{3}=\{[0.5,0.7]\}$, and ${\tilde{h}}_{4}=\{[0.2,0.3],[0.4,0.5]\}$ be four IVHFEs, let the BUM function be *Q*(*x*) = *x* ^2^, and the relative weight vector of the criteria is *ω* = (*ω*_1_, *ω*_2_, *ω*_3_, *ω*_4_) = (0.1,0.3,0.2,0.4).

First, $f_{G}^{A}({\tilde{h}}_{1})=\{0.569,0.834\}$, $f_{G}^{A}({\tilde{h}}_{2})=\{0.334,0.434,0.534\}$, and $f_{G}^{A}({\tilde{h}}_{3})=\{0.569\}$, $f_{G}^{A}({\tilde{h}}_{4})=\{0.234,0.434\}$, since $s(f_{G}^{A}({\tilde{h}}_{1}))=0.702>s(f_{G}^{A}({\tilde{h}}_{3}))=0.569>s(f_{G}^{A}({\tilde{h}}_{2}))$ $=0.434>s(f_{G}^{A}({\tilde{h}}_{4}))=0.334$, then *w*_*ρ*(1)_ = 0.06, *w*_*ρ*(2)_ = 0.31, *w*_*ρ*(3)_ = 0.19, and *w*_*ρ*(4)_ = 0.44, thus

(82) SWC-HFOWA (h~1,h~2,h~3,h~4)=⋃fQA(γ~j)∈fQA(h~j),j=1,2,3,4{1−∏j=1n(1−fQA(γ~j))wρ(j)ωj/∑j=1nwρ(j)ωj}={0.322,0.335,0.354,0.366,0.391,0.402,0.428,0.439,  0.455,0.465,0.486,0.495},WC-HFOWA(h~1,h~2,h~3,h~4)=⋃fQA(γ~j)∈fQA(h~j),j=1,2,3,4{1−∏j=1n(1−fQA(γ~j))wρ(j)ωj/∑j=1nwρ(j)ωj}={0.382,0.411,0.438,0.445,0.452,0.465,0.478,0.495,  0.502,0.508,0.526,0.553},OWC-HFOWA(h~1,h~2,h~3,h~4)=⋃fQA(γ~j)∈fQA(h~j),j=1,2,3,4{1−∏j=1n(1−fQA(γ~j))wρ(j)ωj/∑j=1nwρ(j)ωj}={0.365,0.396,0.431,0.400,0.430,0.444,0.463,0.471,  0.475,0.502,0.501,0.53}.

Furthermore, according to the score function of HFE, we can derive the score values of the aggregated results: $s(\text{SWC-HFOWA}({\tilde{h}}_{1},{\tilde{h}}_{2},{\tilde{h}}_{3},{\tilde{h}}_{4}))=0.412$, $s(\text{WC-HFOWA}\;({\tilde{h}}_{1},{\tilde{h}}_{2},{\tilde{h}}_{3},{\tilde{h}}_{4}))=0.471$, and $s(\text{OWC-HFOWA}({\tilde{h}}_{1},{\tilde{h}}_{2},{\tilde{h}}_{3},{\tilde{h}}_{4}))=0.451$.

From Propositions 39 and 44 and Example 47, we know the main advantage of the SWC-HFOWA operator is that it generalizes both the WC-HFOWA operator and the OWC- HFOWA operator, and it reflects the importance of both the considered argument and its ordered position.

## 5. An Approach to Multiple Criteria Decision Making under the Interval-Valued Hesitant Fuzzy Setting

In this section, we consider the multiple criteria decision making (MCDM) problem where all the criteria values are expressed in interval-valued hesitant fuzzy information. The following notations are used to depict the considered problem. Let *A* = {*A*_1_, *A*_2_,…, *A*_*m*_} be a set of *m* alternatives, let *C* = {*C*_1_, *C*_2_,…, *C*_*n*_} be a set of *n* criteria, and let *ω* = (*ω*_1_, *ω*_2_,…, *ω*_*n*_) be the relative weight vector of criteria, with *ω*_*j*_ ∈ [0,1] and ∑_*j*=1_^*n*^ *ω*_*j*_ = 1. The decision makers provide all the possible values for the alternative *A*_*i*_ against the criterion *C*_*j*_, and represent as the IVHFEs ${\tilde{h}}_{ij}^{\prime }={⋃}_{{\tilde{\gamma }}_{ij}^{\prime }\in {\tilde{h}}_{ij}^{\prime }}\left\{{\tilde{\gamma }}_{ij}^{\prime }=[{\tilde{\gamma }}_{ij}^{\prime L},{\tilde{\gamma }}_{ij}^{\prime U}]\right\}$  (*i* = 1,2,…, *m*, *j* = 1,2,…, *n*), which construct the interval-valued hesitant fuzzy decision matrix ${\tilde{H}}^{\prime }={\left({\tilde{h}}_{ij}^{\prime }\right)}_{m\times n}$.

In general, there are benefit criteria (the bigger the criteria values, the better) and cost criteria (the smaller the criteria values, the better) in MCDM problems. In order to measure all criteria in dimensionless units and to facilitate intercriteria comparisons, in the following we normalize the decision matrix ${\tilde{H}}^{\prime }={\left({\tilde{h}}_{ij}^{\prime }\right)}_{m\times n}$ into a corresponding decision matrix $\tilde{H}={\left({\tilde{h}}_{ij}\right)}_{m\times n}$:

(83) h~ij=⋃γ~ij∈h~ij{γ~ij}={γ~ij′,for  benefit  criteria  Cjγ~ij′c,for  cost  criteria  Cj(i=1,2,…,m,  j=1,2,…,n),

where ${\tilde{\gamma }}_{ij}^{\prime c}$ is the complement of ${\tilde{\gamma }}_{ij}^{\prime }$ such that ${\tilde{h}}_{ij}^{\prime c}={⋃}_{{\tilde{\gamma }}_{ij}^{\prime }\in {\tilde{h}}_{ij}^{\prime }}\{[1-{\tilde{\gamma }}_{ij}^{\prime U},1-{\tilde{\gamma }}_{ij}^{\prime L}]\}.$

In the following, we apply the above synergetic weighted C-HFOW operators to multiple criteria decision making under interval-valued hesitant fuzzy setting.

**Step 1**

Normalize the original interval-valued hesitant fuzzy decision matrix ${\tilde{H}}^{\prime }={\left({\tilde{h}}_{ij}^{\prime }\right)}_{m\times n}$ by (83) and then obtain the normalized interval-valued hesitant fuzzy decision matrix $\tilde{H}={\left({\tilde{h}}_{ij}\right)}_{m\times n}$.

**Step 2**

Select a BUM function *Q*(*x*) according to the DMs' risk preferences [4, 43] and calculate the associated weight vector *w* = (*w*_1_, *w*_2_, ..., *w*_*n*_) by the following formula:

(84) $$\begin{array}{c} w_{j}=Q\left(\frac{j}{n}\right)-Q\left(j-\frac{1}{n}\right)\;\left(j=1,2,\ldots ,n\right). \end{array}$$

**Step 3**

Aggregate decision information of ${\tilde{h}}_{ij}\;(i=1,2,\ldots ,m,j=1,2,\ldots ,n)$ and obtain the HFEs *h*_*i*_  (*i* = 1,2,…, *m*) for the alternatives *A*_*i*_  (*i* = 1,2,…, *m*) by the SWC-HFOWA operator:

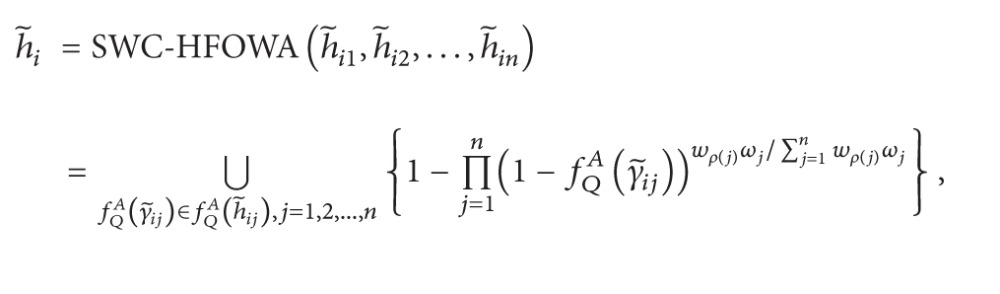
(85)
where

(86) fQA(γ~ij) =⋃γ~ij∈h~ij(1−exp⁡(∫01dQ(x)dx×ln⁡⁡[1−γ~ijU−(γ~ijU−γ~ijL)x]dx)),

or the SWC-HFOWG operator

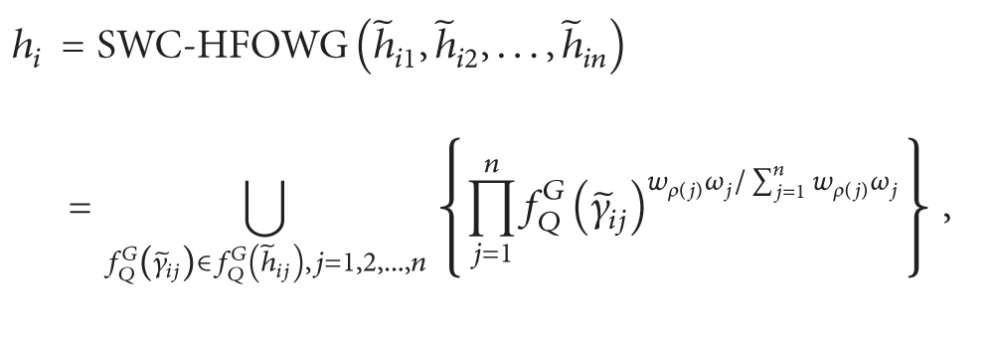
(87)
where

(88) fQG(h~ij) =⋃γ~ij∈h~ij(exp⁡(∫01dQ(x)dxln⁡⁡[1−γ~ijL(γ~ijUγ~ijL)x]dx)).

**Step 4**

Calculate the score values *s*(*h*_*i*_)  (*i* = 1,2,…, *m*) of *h*_*i*_  (*i* = 1,2,…, *m*) by Definition 4:

(89) $$\begin{array}{c} S\left(h_{i}\right)=\frac{1}{\#h_{i}}\underset{\gamma_{i}\in h_{i}}{\sum }\gamma_{i},\;i=1,2,\ldots ,m. \end{array}$$

**Step 5**

Rank all the alternatives *A*_*i*_  (*i* = 1,2,…, *m*) according to *S*(*h*_*i*_)  (*i* = 1,2,…, *m*) in descending order.

## 6. Illustrative Example

Service activities have become the fundamental and dominant factors of the economic system over the past decades and the significance and influence of service quality have been recognized through the great effect on customer satisfaction and loyalty. Relevant studies indicated that service quality is a key factor for survival and development in today's keen competition. Thus, the evaluation of service quality has become an important issue. Suppose that there are five alternatives (high-tech enterprises) *A*_*i*_  (*i* = 1,2,…, 5) participating to the evaluation of service quality according to four main criteria: *C*_1_: reliability, *C*_2_: responsiveness, *C*_3_: attitude, and *C*_4_: speed, the relative weight vector of the criteria is *ω* = (*ω*_1_, *ω*_2_, *ω*_3_, *ω*_4_) = (0.1,0.4,0.2,0.3). Several DMs are invited to form a committee and evaluate the service quality of the five alternatives. The results evaluated by the DMs are contained in an interval-valued hesitant fuzzy decision matrix, shown in Table 1.

**Step 1**

Since all the criteria are the benefit criteria, then the criteria values do not need normalization.

**Step 2**

Select a BUM function *Q*(*x*) = *x* ^2^ according to the DMs' risk preferences, and calculate the associated weight vector *w* = (*w*_1_, *w*_2_, *w*_3_, *w*_4_) by (84). The calculated associated weights are listed as follows:

(90) w1=(14)2−(04)2=0.06,  w2=(24)2−(14)2=0.19,w3=(34)2−(24)2=0.31,  w4=(44)2−(34)2=0.44.

**Step 3**

Utilize the SWC-HFOWA operator, (85) and (86), to obtain the *h*_*i*_  (*i* = 1,2,…, *m*) for the alternatives *A*_*i*_  (*i* = 1,2,…, *m*).

First, we use the C-HFOWA to aggregate each IVHFE, and the aggregated results are listed in Table 2.

Since $s\left(f_{G}^{A}\left({\tilde{h}}_{11}\right)\right)=0.634>s\left(f_{G}^{A}\left({\tilde{h}}_{13}\right)\right)=0.434>s\left(f_{G}^{A}\left({\tilde{h}}_{12}\right)\right)=0.369>s\left(f_{G}^{A}\left({\tilde{h}}_{14}\right)\right)=0.334$, then *w*_*ρ*(1)_ = 0.06, *w*_*ρ*(2)_ = 0.31, *w*_*ρ*(3)_ = 0.19, and *w*_*ρ*(4)_ = 0.44.

Thus

(91) h1=SWC−HFOWA(h~11,h~12,h~13,h~14)=⋃fQ(γ~1j)∈fQ(h~1j),j=1,2,3,4{1−∏j=14((1−fQ(γ~1j))wρ(j)ωj/∑j=14wρ(j)ωj}={0.327,0.334,0.410,0.417}.

Similarly, we can obtain

(92) h2=SWC-HFOWA(h~21,h~22,h~23,h~24)={0.428,0.468,0.485,0.520,0.545,0.576},h3=SWC-HFOWA(h~31,h~32,h~33,h~34)={0.297,0.326,0.369,0.395,0.490,0.512,0.543,0.562},h4=SWC-HFOWA(h~41,h~42,h~43,h~44)={0.465,0.499,0.644,0.667},h5=SWC-HFOWA(h~51,h~52,h~53,h~54)={0.409,0.415,0.506,0.510}.

**Step 4**

Calculate the scores values of *h*_*i*_  (*i* = 1,2,…, 5) by (89):

(93) s(h1)=0.372,  s(h2)=0.504,  s(h3)=0.437,s(h4)=0.569,  s(h5)=0.46.

**Step 5**

Rank the alternatives *A*_*i*_ according to the score values *s*(*h*_*i*_)  (*i* = 1,2,…, 5); the ranking results are *A*_4_≻*A*_2_≻*A*_5_≻*A*_3_≻*A*_1_.

In the following, we use the SWC-HFOWG operator to solve the same problem.

*Step  1*′. This step is the same as the above Step 1.

*Step  2*′. This step is the same as the above Step 2.

*Step  3*′. Utilize the SWC-HFOWG operator, (87) and (88), to obtain the *h*_*i*_  (*i* = 1,2,…, *m*) for the alternatives *A*_*i*_  (*i* = 1,2,…, *m*).

First, utilize C-HFOWG operator to aggregate each IVHFE, and the aggregated results are listed in Table 3.

Then, obtain the aggregated results

(94) h1=SWC-HFOWG(h~11,h~12,h~13,h~14)={0.303,0.400,0.305,0.403},h2=SWC-HFOWG(h~21,h~22,h~23,h~24)={0.386,0.458,0.523,0.414,0.491,0.561},h3=SWC-HFOWG(h~31,h~32,h~33,h~34)={0.272,0.305,0.289,0.323,0.461,0.516,0.489,0.547},h4=SWC-HFOWG(h~41,h~42,h~43,h~44)={0.436,0.589,0.447,0.605},h5=SWC-HFOWG(h~51,h~52,h~53,h~54)={0.377,0.468,0.377,0.469}.

*Step  4*′. Calculate the score values of *h*_*j*_  (*j* = 1,2,…, 5) by (89):

(95) s(h1)=0.353,  s(h2)=0.472,  s(h3)=0.400,s(h4)=0.519,  s(h5)=0.423.

*Step  5*′. Rank the alternatives *A*_*i*_  (*i* = 1,2,…, 5) according to the score values *s*(*h*_*i*_)  (*i* = 1,2,…, 5); the ranking results are *A*_4_≻*A*_2_≻*A*_5_≻*A*_3_≻*A*_1_.

Obviously, the identical ranking results can be obtained through the C-HFOWA operator based and the C-HFOWG operator based approaches, which implies the two proposed approaches all are feasible and effective.

Moreover, to understand more the effect of different types of weights in aggregation, we use the WC-HFOWA, OWC-HFOWA, and SWC-HFOWA operators to the example above and their final score values and ranking results are listed in Table 4.

From Table 4, it is clear that despite the score values obtained by the WC-HFOWA, OWC-HFOWA and SWC-HFOWA operators are different and the ranking results of the alternatives derived from them are the same; that is, *A*_4_≻*A*_2_≻*A*_5_≻*A*_3_≻*A*_1_. The reasons about the difference of score values are intuitive that, as discussed above, the WC-HFOWA operator focuses solely on the relative weights and ignores the associated weights, while the OWC-HFOWA operator focuses only on the associated weights and ignores the relative weights. The SWC-HFOWA operator comprehensively considers both the associated weights and the relative weights. Hence, the results derived by SWC-HFOWA operator are more feasible and effective and the identical ranking results imply that the WC-HFOWA, OWC-HFOWA, SWC-HFOWA, and WC-HFOWG all are effective and reasonable.

Furthermore, we compare our proposed operators with the existing interval-valued hesitant fuzzy aggregation operators; here we use the IVHFWA operator [11] to the above example and the aggregated results are listed as follows.

(96) h~1={[0.317,0.459],[0.351,0.495],[0.373,0.511],  [0.405,0.544]},h~2={[0.507,0.666],[0.534,0.695],[0.537,0.689],[0.562,0.716],[0.569,0.716],[0.593,0.741]},h~3={[0.302,0.428],[0.421,0.654],[0.347,0.473], [0.459,0.681],[0.333,0.536],[0.448,0.719], [0.377,0.572],[0.484,0.741]}h~4={[0.454,0.662],[0.502,0.697],[0.611,0.806],  [0.645,0.826]},h~5={[0.425,0.557],[0.448,0.587],[0.497,0.664],  [0.517,0.687]}.

Then we calculate the score values of ${\tilde{h}}_{j}\;(j=1,2,\ldots ,5)$ according to Definition 8.

$s({\tilde{h}}_{1})=[0.362,0.502]$, $s({\tilde{h}}_{2})=[0.550,0.704]$, $s({\tilde{h}}_{3})=[0.396,0.600]$, $s({\tilde{h}}_{4})=[0.553,0.748]$, and $s({\tilde{h}}_{5})=[0.472,0.624]$. Since the score values of  ${\tilde{h}}_{j}\;(j=1,2,\ldots ,5)$ are still interval-valued form, to rank these score values, we have to first compare each pair of score values of ${\tilde{h}}_{j}\;(j\in 1,2,\ldots ,5)$ by using the possibility degree formula, for example, the possibility degree of $s({\tilde{h}}_{1})\geq s({\tilde{h}}_{2})$:

(97) P(s(h~1)≥s(h~2))=max⁡{1−max⁡(0.704−0.362(0.502−0.362)+(0.704−0.550),0),0}=0.123.

Similarly, we calculate the rest possibility degrees and then obtain a possibility degree matrix

(98) $$\begin{array}{c} \begin{array}{c} P=\left(\begin{array}{cc} \begin{array}{cc} 0.5, & 0.123,\\ 0.877, & 0.5, \end{array} & \begin{array}{ccc} 0, & 0.117, & 0\\ 1, & 0, & 1 \end{array}\\ \begin{array}{cc} 1, & 0,\\ 0.883, & 1,\\ 1, & 0, \end{array} & \begin{array}{ccc} 0.5, & 0, & 0\\ 1, & 0.5, & 1\\ 1, & 0, & 0.5 \end{array} \end{array}\right) \end{array}. \end{array}$$

Finally, we average all elements in each line of the possibility degree matrix and then get the relative possibility degrees *p*_*i*_  (*i* = 1,2,…, *m*) of the alternatives *A*_*i*_  (*i* = 1,2,…, *m*) and rank the alternatives *A*_*i*_  (*i* = 1,2,…, *m*) according to the relative possibility degrees *p*_*i*_  (*i* = 1,2,…, 5). The ranking results are *p*_4_ = 0.877≻*p*_2_ = 0.675≻*p*_5_ = 0.5≻*p*_3_ = 0.3≻*p*_1_ = 0.148.

By the above analysis, we can find that the final decision results (score values) are different and yet the ranking results of alternatives derived from the WC-HFOWA, OWC-HFOWA, SWC-HFOWA, WC-HFOWG, and IVHFWA operators are identical, which further indicate that they all are effective and reasonable.

1. The IVHFWA operator is straightforward extensions of HFWA operator; it only focuses on the endpoints of the closed intervals of IVHFEs and therefore is not rich enough to capture all the information contained in IVHFEs and much useful information may be lost. However our operators aggregate all the information over closed intervals of IVHFEs and thus can effectively avoid the information loss.
2. In decision making with the IVHFWA operator, the score values of aggregated results (alternatives) are still interval-valued. In order to rank the alternatives, we have to first use the possibility degree formula to compare each pair of score values of the alternatives and then calculate the relative possibility degrees of the alternatives. Such procedure needs a large amount of computational efforts and takes a lot of time to be accomplished, especially, with the increases of the number of alternatives. Moreover, if use the IVHFOWA operator [11] or the IVHFHA operator [11] to solve decision making problems, the process of calculation will be more complex because the IVHFEs to be aggregated require to be reordered before the aggregation. What is more, the relative possibility degrees of alternatives are only relative compared values rather than the real performances of alternatives, thus they have no meaning in reality. However our operators and approach can directly derive the results which take the form as HFE and the precise score values, respectively, and thus efficiently avoid the complex comparisons and rankings. Therefore, the computational complexity of our operators and approaches is much lower than the interval-valued hesitant fuzzy aggregation operators. Additionally, our approaches can rank the alternatives by directly using their score values and they are much more interpretable.
3. The aggregation of IVHFWA operator does not consider the DMs' risk preferences, which implies that the importance of all information in the closed intervals of IVHFEs is the same. However, the decision result needs usually to reflect the DMs' risk preferences, that is to say, the DMs' risk preferences should be added to the aggregation of each possible interval of IVHFEs but the endpoints of the possible intervals of IVHFEs should not be simply regarded as the same. Our operators and approaches consider the DMs' risk preferences via the basic unit-interval monotonic (BUM) function, which are very suitable for the practical decision making situations.

## 7. Conclusion

To efficiently and effectively aggregate the interval-valued hesitant fuzzy information, in this paper, we have presented some continuous hesitant fuzzy aggregation operators, that is, the continuous hesitant fuzzy ordered weighted averaging (C-HFOWA) operator and the continuous hesitant fuzzy ordered weighted geometric (C-HFOWG) operator, and their fundamental properties are studied in detail. Then, we extended the operators to aggregate multiple interval-valued hesitant fuzzy elements and then developed the weighted C-HFOW (WC-HFOWA and WC-HFOWG), ordered weighted C-HFOW (OWC-HFOWA and OWC-HFOWG), and synergetic weighted C-HFOW (SWC-HFOWA and SWC-HFOWG) operators; some properties of them are also discussed. Based on the SWC-HFOW operators, we developed an approach for multicriteria decision making under interval-valued hesitant fuzzy setting. Finally, a practical example involving the evaluation of service quality of high-tech enterprises is carried out and some comparative analysis are performed to illustrate the applicability and effectiveness of the developed approach. In the future, we will further investigate the continuous hesitant fuzzy aggregation operators that there is some degree of interdependent characteristics between argument variables with the help of the continuous Choquet integral [44, 45].

## Figures and Tables

**Table 1:** Interval-valued hesitant fuzzy decision matrix.

	*C*_1_	*C*_2_	*C*_3_	*C*_4_
*A*_1_	{[0.5,0.6],[0.7,0.8]}	{[0.3,0.5]}	{[0.4,0.5]}	{[0.2,0.3], [0.4,0.5]}
*A*_2_	{[0.3,0.5],[0.6,0.8]}	{[0.3,0.4],[0.4,0.5],[0.5,0.6]}	{[0.8,0.9]}	{[0.5,0.7]}
*A*_3_	{[0.6,0.8]}	{[0.2,0.3],[0.5,0.8]}	{[0.3,0.4],[0.5,0.6]}	{[0.3,0.4],[0.4,0.7]}
*A*_4_	{[0.5,0.7],[0.8,0.9]}	{[0.3,0.6],[0.7,0.9]}	{[0.7,0.8]}	{[0.4,0.6]}
*A*_5_	{[0.7,0.8],[0.8,0.9]}	{[0.3,0.4],[0.5,0.7]}	{[0.6,0.7]}	{[0.3,0.5]}

**Table 2:** Aggregated results derived by C-HFOWA operator.

	*C*_1_	*C*_2_	*C*_3_	*C*_4_
*A*_1_	{0.534, 0.734}	{0.369}	{0.434}	{0.234, 0.434}
*A*_2_	{0.369, 0.669}	{0.334, 0.434, 0.534}	{0.834}	{0.569}
*A*_3_	{0.669}	{0.234, 0.603}	{0.334, 0.534}	{0.334, 0.503}
*A*_4_	{0.569, 0.834}	{0.403, 0.769}	{0.734}	{0.469}
*A*_5_	{0.734, 0.834}	{0.334, 0.569}	{0.634}	{0.369}

**Table 3:** Aggregated results derived by C-HFOWG operator.

	*C*_1_	*C*_2_	*C*_3_	*C*_4_
*A*_1_	{0.531, 0.732}	{0.356}	{0.431}	{0.229, 0.431}
*A*_2_	{0.356, 0.66}	{0.33, 0.431, 0.531}	{0.832}	{0.559}
*A*_3_	{0.66}	{0.229, 0.585}	{0.33, 0.531}	{0.33, 0.482}
*A*_4_	{0.559, 0.832}	{0.378, 0.761}	{0.732}	{0.458}
*A*_5_	{0.732, 0.832}	{0.33, 0.559}	{0.632}	{0.356}

**Table 4:** Score values and ranking results derived by WC-HFOWA, OWC-HFOWA, and SWC-HFOWA operators.

	*A*_1_	*A*_2_	*A*_3_	*A*_4_	*A*_5_	Ranking results
WC-HFOWA	0.409	0.601	0.463	0.619	0.523	*A*_4_≻*A*_2_≻*A*_5_≻*A*_3_≻*A*_1_
SWC-HFOWA	0.372	0.504	0.437	0.569	0.46	*A*_4_≻*A*_2_≻*A*_5_≻*A*_3_≻*A*_1_
OWC-HFOWA	0.390	0.532	0.450	0.594	0.492	*A*_4_≻*A*_2_≻*A*_5_≻*A*_3_≻*A*_1_
